# Crystal structure solution and high-temperature thermal expansion in NaZr_2_(PO_4_)_3_-type materials

**DOI:** 10.1107/S2052520624001598

**Published:** 2024-03-22

**Authors:** Benjamin S. Hulbert, Julia E. Brodecki, Waltraud M. Kriven

**Affiliations:** a Materials Science and Engineering, University of Illinois at Urbana-Champaign, 1304 W. Green St, Urbana, Illinois 61801, USA; Moscow State University, Russian Federation

**Keywords:** NZP-type materials, CaZr_4_(PO_4_)_6_, SrZr_4_(PO_4_)_6_, phase transformation, thermal expansion, structure solution, Fourier difference map, powder diffraction

## Abstract

The NaZr_2_P_3_O_12_ family of materials have shown low and tailorable thermal expansion properties. In this study, SrZr_4_P_6_O_24_ (SrO·4ZrO_2_·3P_2_O_5_), CaZr_4_P_6_O_24_ (CaO·4ZrO_2_·3P_2_O_5_), MgZr_4_P_6_O_24_ (MgO·4ZrO_2_·3P_2_O_5_), NaTi_2_P_3_O_12_ [½(Na_2_O·4TiO_2_·3P_2_O_5_)], NaZr_2_P_3_O_12_ [½(Na_2_O·4ZrO_2_·3P_2_O_5_)], and related solid solutions were synthesized using the organic–inorganic steric entrapment method. The samples were characterized by *in-situ* high-temperature X-ray diffraction from 25 to 1500°C at the Advanced Photon Source and National Synchrotron Light Source II. The average linear thermal expansion of SrZr_4_P_6_O_24_ and CaZr_4_P_6_O_24_ was between −1 × 10^−6^ per °C and 6 × 10^−6^ per °C from 25 to 1500°C. The crystal structures of the high-temperature polymorphs of CaZr_4_P_6_O_24_ and SrZr_4_P_6_O_24_ with *R* 3 *c* symmetry were solved by Fourier difference mapping and Rietveld refinement. This polymorph is present above ∼1250°C. This work measured thermal expansion coefficients to 1500°C for all samples and investigated the differences in thermal expansion mechanisms between polymorphs and between compositions.

## Abstract 2

The crystal structure in space group *R* 3 *c* in a high-temperature polymorph of SrZr_4_P_6_O_24_ and CaZr_4_P_6_O_24_ was solved with Fourier difference mapping. The thermal expansion tensors for several NaZr_2_P_3_O_12_-type materials were measured from 25 to 1500°C and crystallographic thermal expansion mechanisms investigated.

## 1. Introduction

NaZr_2_P_3_O_12_ [‘NZP’ or ½(Na_2_O·4ZrO_2_·3P_2_O_5_)] and structurally related materials have been studied primarily for their thermal expansion (Lind, 2012 ▸; Shi *et al.*, 2021 ▸; Evans, 1999 ▸; Barrera *et al.*, 2005 ▸; Takenaka, 2018 ▸; Romao *et al.*, 2013 ▸; Dove & Fang, 2016 ▸; Breval & Agrawal, 1995 ▸; Roy *et al.*, 1989 ▸) and ionic conductivity properties (Goodenough *et al.*, 1976 ▸; Rao *et al.*, 2021 ▸). The NZP structure was first solved by Hagman & Kierkegaard (1968 ▸). A study by Boilot *et al.* (1979 ▸) of the related solid solution Na_l+*x*_Zr_2_Si_*x*_P_3–*x*_O_12_ (where 0 < *x* < 3) showed low thermal expansion near *x* = 0, and high ionic conductivity near *x* = 2. Since the 1980s, many cation substitutions in this NZP structure have been found and studied, leading to a class of materials called NZP-type materials (Breval & Agrawal, 1995 ▸; Limaye *et al.*, 1987 ▸, 1991 ▸; Agrawal, 1996 ▸; Agrawal & Stubican, 1985 ▸; Pet’kov & Asabina, 2004 ▸; Govindan Kutty *et al.*, 1998 ▸, 1994 ▸; Miyazaki *et al.*, 2008 ▸).

NZP-type material properties have been explored for their low and tailorable thermal expansion, including thermal shock resistance, substrates for optical applications, and thermal barrier coatings (Trice *et al.*, 1999 ▸). Additionally, there has been research into their use in energy storage/solid state electrolyte applications due to the high ionic conductivity of certain compositions (Goodenough *et al.*, 1976 ▸; Rao *et al.*, 2021 ▸). For this reason, they have been called NaSiCON (Na super-ionic conductors) materials (Zhang *et al.*, 2023 ▸; Li *et al.*, 2022 ▸; Aono & Suginoto, 1996 ▸). The ability to incorporate a range of cations into the NZP framework structure and its high-temperature stability has led to its investigation for use as an actinide host material for the containment of spent fuel from nuclear reactors (Gregg *et al.*, 2013 ▸; Krishnaiah *et al.*, 2003 ▸). NZP-type materials have also been studied for their electrical and thermal conductivity applications (Trice *et al.*, 1999 ▸; Gregg *et al.*, 2013 ▸; Krishnaiah *et al.*, 2003 ▸; Liu *et al.*, 1994 ▸) as well as for use in gas sensors (Dang & Guo, 2011 ▸; Kida *et al.*, 2011 ▸).

The NZP crystal structure can have many substitutions for each cation in its unit cell (Pet’kov & Orlova, 2003 ▸; Alamo, 1993 ▸; Breval *et al.*, 2000 ▸; Breval & Agrawal, 1995 ▸). In NZP, the Na^+^ cation that resides on the *M*1 and *M*2 sites can have substitutions by most Group I or II elements, as well as many transition metals and rare earth elements. The Zr^4+^ cation on the *R* site can have substitutions by Ge^4+^, Sn^4+^, Ti^4+^, Hf^4+^ and Nb^5+^ cations as well as partial occupancy of several other elements. The P^5+^ cation that resides on the P-site can have substitutions with Si^4+^ and S^6+^. Having a wide range of substitutions while maintaining a similar crystal structure allows for the opportunity to tailor the material property of interest. In the Na_4_Zr_2_(SiO_4_)_3_ structure there are four times as many Na^+^ sites as in NaZr_2_(PO_4_)_3_. In Na_4_Zr_2_(SiO_4_)_3_, instead of residing only between two ZrO_6_ octahedra, there are additional *M* sites between ZrO_6_ octahedra and SiO_4_ tetrahedra. A description of all the *M* sites can be found elsewhere (Qui *et al.*, 1981 ▸) and some of these sites are shown in Fig. S1 (supporting information). This wide variation in number of cations and their valence charges in the NZP-type structure has resulted in numerous studies (Breval & Agrawal, 1995 ▸; Limaye *et al.*, 1987 ▸, 1991 ▸; Agrawal, 1996 ▸; Agrawal & Stubican, 1985 ▸). These studies probed the changes in physical properties due to variations in the NZP lattice structure and its accommodation of different cations on each of the *M*, *R* and *P* sites.

NZP-type materials form several different variations of the standard NZP crystal structure based on composition and synthesis methods, leading to crystallization in the following space groups: 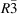 , 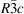 , *R*32, *Pbcn*, 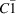 and *P*2_1_/3 (Pet’kov & Orlova, 2003 ▸). There are many ways in which the symmetry elements are broken or added between these different space groups. Generally, these structural changes depend on whether there is an atom:

(i) on the *M*1 site,

(ii) on *M*1, *M*2 or one of the other six common interstitial sites,

(iii) on none of the *M* sites,

(iv) partially occupying *M* and *R* sites with another element,

(v) if the *R*-site octahedron begins to rotate off the *c* unit-cell axis (for the monoclinic space groups), or

(vi) if there is a change in valence charge for the *M*, *R* and/or *P* sites (often changing the occupancy to satisfy charge balance).

In the past, there has been disagreement over the correct crystal structure for some NZP-type materials (Senbhagaram *et al.*, 1989 ▸), which are sometimes differentiated by several relatively small XRD peaks.

Past thermal expansion studies on NZP-type materials covered the temperature range of 25 to 500°C for most compositions. Few samples have been studied over 1000°C (Breval & Agrawal, 1995 ▸). The melting point of these materials was shown to be between 1600 to 1900°C (Agrawal, 1996 ▸; Pet’kov & Asabina, 2004 ▸), suggesting the possibility that they may be used over a wide range of temperatures. However, use of NZP-type materials between 1000°C and their melting point would require substantial extrapolation of phase stability and thermal expansion from lower-temperature crystallographic data reported in the literature.

**Figure 1:**
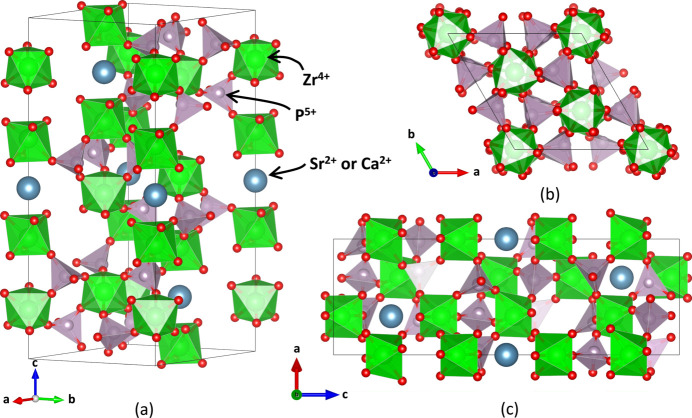
Crystal structure for CaZr_4_P_6_O_24_ (CaZP) at 25°C in 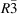 (space group No. 148): (*a*) showing the corner-sharing nature of ZrO_6_ octahedra and PO_4_ tetrahedra seen in NZP-type materials, with alternate views (*b*) along the *c* axis and (*c*) along the *b* axis. SrZr_4_P_6_O_24_ (SrZP) is isostructural, having the same space group, similar unit-cell lengths and atom positions.

One of the variations on the NZP-type structure that was studied here is CaZr_4_P_6_O_24_ (CaO·4ZrO_2_·3P_2_O_5_ or ‘CaZP’), the crystal structure of which at 25°C is depicted in Fig. 1 ▸. It consists of a network structure of corner-sharing PO_4_ tetrahedra and ZrO_6_ octahedra. Ca^2+^ occupies the site between two ZrO_6_ octahedra along the *c* axis and is six-coordinated. The P—O and Zr—O bonds are more rigid and have relatively minor increases in length with temperature, whereas the Ca—O bond is weaker and has more expansion with temperature (Khosrovani & Sleight, 1999 ▸; Hazen & Prewitt, 1977 ▸). For this reason, NZP-type materials have sometimes been studied by treating the PO_4_ tetrahedra and ZrO_6_ octahedra each as rigid units (Tao & Sleight, 2003 ▸; Khosrovani & Sleight, 1999 ▸). Coordinated vibrational movements of rigid polyhedra can be described in terms of the rigid-unit-modes (RUM) characteristic of their thermal expansion behavior (Tao & Sleight, 2003 ▸; Liang *et al.*, 2008 ▸). In structures with corner-sharing polyhedra, the static structural distortions of metal–oxygen–metal (*M*–O–*M*) angles, or bridging-oxygen angles, are measured to describe the way in which sets of connected polyhedra rotate. The rotation of polyhedra relative to the unit-cell axes have also been measured to describe thermal expansion mechanisms in related systems (Alamo, 1993 ▸; Lenain *et al.*, 1987 ▸). Unit-cell diagrams for NaZr_2_(PO_4_)_3_ and Na_4_Zr_2_(SiO_4_)_3_ illustrating the differences in interstitial site vacancy and polyhedron connectivity related to the *P* site can be found in Fig. S1.

**Figure 2:**
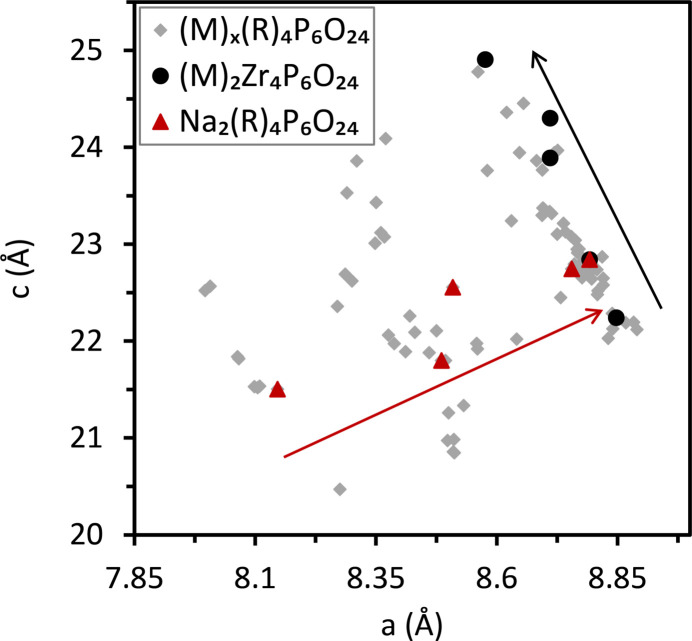
The *c* versus *a* unit-cell parameters for several NZP-type materials with substitutions on the *M* and *R* sites. The filled black circles show the effect of substitution of the *M* (*M*1 and/or *M*2) site cation in (*M*)_2_Zr_4_P_6_O_24_, where *M* = Li, Na, K, Rb and Cs. The red triangles show the effect of substitution of the *R* site cation in Na_2_(*R*)_4_P_6_O_24_, where *R* = Ge, Ti, Sn, Zr and Hf. The black and red arrows indicate the directions of increasing size of atomic radii for the *M* and *R* sites, respectively. Tabulated unit-cell parameter values from the ICDD database (Kabekkodu *et al.*, 2002 ▸) are given in Table S4.

The effect of *M*1-, *M*2- and *R*-site substitutions on unit-cell parameters is shown in Fig. 2 ▸ for the 25°C structures in the hexagonal setting for many NZP-type materials. Larger cations on the *M*1/*M*2 site cause the *a* and *b* unit-cell axes (which are equal in the hexagonal setting) to contract and the *c* unit-cell axis to expand. Larger cations on the *R* site cause both the *a* and *c* axes to expand. Examining the effect of cation substitutions at 25°C can begin to explain directions and modes by which the NZP-type network structure can accommodate changes. The data in Fig. 2 ▸ are from PDF card entries in the ICDD-PDF 4+ database (ICDD v. 2022, International Center for Diffraction data, Newton Square, PA, USA) (Kabekkodu *et al.*, 2002 ▸) which are tabulated in Table S4.

In contrast to the unit-cell parameters at 25°C, thermal expansion values do not always follow similar trends with cation substitutions. Alamo (1993 ▸) described a trend in the NZP-type materials in which there is differing thermal expansion in the *a* unit-cell axis depending on crystal symmetry. It was shown that NZP-type materials which crystallize in space group 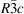 exhibit negative thermal expansion of the *a* unit-cell axis on heating, whereas those which crystallize in space group 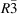 show positive expansion. Both space groups exhibit expansion along the *c* axis on heating. However, CaZP does not follow this trend and crystallizes in space group 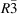 and shows contraction along the *a* axis. Past studies of this material have not determined what causes this anomaly (Pet’kov *et al.*, 2002 ▸; Woodcock *et al.*, 1999*a* ▸).

Near room-temperature SrZr_4_P_6_O_24_ (SrZP) and CaZP exhibit low positive and low negative thermal expansion, respectively (Limaye *et al.*, 1987 ▸; Breval & Agrawal, 1995 ▸). This difference in thermal expansion is due to thermal expansion along the *a* axis having opposite signs. Several studies (Woodcock *et al.*, 1999*a* ▸; Rashmi & Shrivastava, 2011 ▸; Govindan Kutty *et al.*, 1998 ▸; Wang *et al.*, 2018 ▸; Pet’kov *et al.*, 2002 ▸; Agrawal & Stubican, 1985 ▸; Limaye *et al.*, 1987 ▸; Fischer *et al.*, 2004 ▸; Chakraborty *et al.*, 2005 ▸) have investigated the Ca_1–*x*_Sr_*x*_Zr_4_P_6_O_24_ solid solution to determine the solid solution range with approximately zero thermal expansion. However, the *x*-value and temperature range is not agreed upon. The reason for this unexpected difference in thermal expansion along the *a*-axis has still not been determined, which is surprising given that these materials, each having two cations, Sr^2+^ and Ca^2+^, possess similar ionic radii and valence charges. A study (Woodcock *et al.*, 1999*a* ▸) investigated Ca_0.5_Sr_0.5_Zr_4_P_6_O_24_ by neutron powder diffraction from 25 to 800°C, including the unusual behavior of the *a*-axis expansion. It is unclear if there are compositions in the Ca_1–*x*_Sr_*x*_Zr_4_P_6_O_24_ solid solution that have near-zero thermal expansion at temperatures above 800°C. Past research has been unable to determine a difference in the thermal expansion mechanism explaining how crystal structures accommodate these different thermal expansions along the *a* axis.

NaTi_2_P_3_O_12_ (NaTP) has been studied for its promising properties and application as an anode material in Na-ion batteries (Wu *et al.*, 2019 ▸). NaTP exhibits the usual thermal expansion for NZP-type materials that crystallize in space group 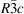 . It has a negative value along the *a* axis and positive value along the *c* axis. However, a recent study (Ribero *et al.*, 2016 ▸) shows that the *a* axis contraction appears to change direction near 1000°C. If this is indeed the case, NaTP could be a good candidate to understand the thermal expansion mechanism for the differing *a*-axis expansions in NZP-type materials.

The goal in this study was to measure the thermal expansion values of several NZP-type materials from 25 to 1500°C, determine phase stability in this temperature range, solve the high-temperature crystal structures of SrZP and CaZP, probe the differences in mechanisms of thermal expansion between the opposite *a*-axis thermal expansions in SrZP and CaZP, and examine how the thermal expansion changes in high-temperature NaTP. The NZP-type material compositions studied here are SrZr_4_P_6_O_24_, CaZr_4_P_6_O_24_, NaTi_2_P_3_O_12_, NaZr_2_P_3_O_12_ and MgZr_4_P_6_O_24_ (MgZP).

## 2. Experimental

### 2.1. Powder synthesis

**Figure 3:**
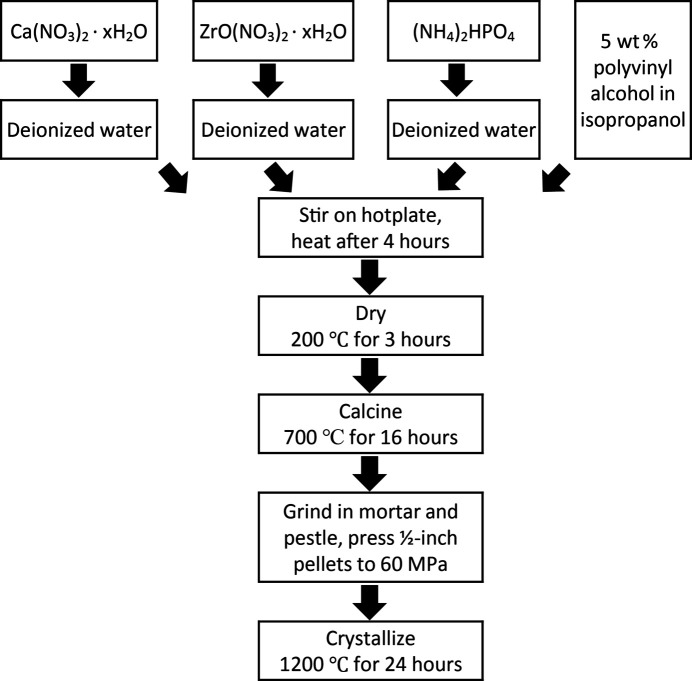
Flow chart for the synthesis of CaZP by the organic–inorganic steric entrapment method. The molar ratios of the precursors are determined by the stoichiometry of the sample.

Ca_1–*x*_Sr_*x*_Zr_4_P_6_O_24_ (where *x* = 0, 0.2, 0.4, 0.5, 0.6, 0.8, 1), Na_2_Zr_4_P_6_O_24_, Na_2_Ti_4_P_6_O_24_ and MgZr_4_P_6_O_24_ powder samples were synthesized via the organic–inorganic steric entrapment method (Nguyen *et al.*, 1999 ▸), as depicted in Fig. 3 ▸ for CaZP. Stoichiometric ratios of calcium nitrate, Ca(NO_3_)_2_·*x*H_2_O, and zirconium oxynitrate hydrate, ZrO(NO_3_)_2_·*x*H_2_O, were mixed in an aqueous solution. For NTP, titanium isopropoxide, Ti[OCH(CH_3_)_2_]_4_, in iso­propanol was the *R*-site cation precursor source. For SrZP, CaZP, MgZP and NTP, strontium nitrate [Sr(NO_3_)_2_·*x*H_2_O] calcium nitrate [Ca(NO_3_)_2_·*x*H_2_O] magnesium nitrate [Mg(NO_3_)_2_·*x*H_2_O] and sodium nitrate (NaNO_3_), respectively, were the precursor chemicals for the *M*-site cation. Then ammonium phosphate dibasic [(NH_4_)_2_HPO_4_] in aqueous solution was added to the solution, dropwise using a pipette. Polyvinyl alcohol (PVA), diluted to 5 wt% in deionized water, was added to achieve a PVA monomer to total cation valence charge ratio of four to one, with the total charge being +48 from the precursors in CaZP for the Ca^2+^, Zr^4+^ and P^5+^ cations. The concentrations of the resulting aqueous solution were not measured after being combined. The amounts of solvent used varied; an unmeasured amount was employed to cleanse the beakers while transferring. The amount of solvent was not measured due to its removal during the first heat treatment step. Additional details describing the steric entrapment method can be found in the following references: Nguyen *et al.* (1999 ▸), Gülgün *et al.* (1999 ▸, 2002 ▸), Ribero & Kriven (2015 ▸).

The solution was stirred for 4 h at 25°C, then heated to evaporate some of the iso­propanol and water, leaving a viscous gel. This was followed by drying for 3 h at 200°C following a 5°C min^−1^ ramp rate of in a Carbolite box furnace to remove any remaining iso­propanol and water. This porous mass was then ground with a mortar and pestle and calcined at 700°C for 16 h after a 5°C min^−1^ ramp rate. After the calcination step the powder was again ground using a mortar and pestle and formed into ∼0.5-inch diameter pellets at a load of ∼60 MPa in a Carver press. These pellets were then set on platinum foil and covered with some sacrificial powder in an alumina crucible and crystallized at 1200°C for 24 h after a 5°C min^−1^ ramp rate.

### 2.2. Preliminary characterization

Successful synthesis of each sample was determined prior to *in-situ* synchrotron experiments by X-ray diffraction (XRD) and X-ray fluorescence (XRF). The crystal structure of the samples was determined by XRD with a Bruker D8 Advance diffractometer, using Cu *K*α radiation (1.5418 A, 40 kV, 30 mA). The crystalline phase was identified with reference to the International Centre for Diffraction Data PDF-4+ database (Kabekkodu *et al.*, 2002 ▸) accessed through *Jade 9.4.1* software (Materials Data Inc., Livermore, CA, USA). X-ray fluorescence (XRF) determined the elemental composition of each sample on a Shimadzu EDX-7000 (Shimadzu America, Chicago, IL, USA) spectrometer by collecting a qualitative scan and a quantitative scan of the elements present.

### 2.3. High-temperature synchrotron powder XRD

*In-situ* XRD experiments were used to study these samples in an optical furnace in capillaries from 25°C to 1600°C. Experiments were conducted with optical furnaces: a Quadrupole Lamp Furnace (QLF) (Sarin *et al.*, 2006 ▸) at beamline 17 BM-B, Advanced Photon Source (APS), Argonne National Laboratory or a Hexapole Lamp Furnace (HLF) at beamline 28-ID-2 (Shi *et al.*, 2013 ▸), National Synchrotron Light Source II, Brookhaven National Laboratory. The QLF is composed of four halogen bulbs, whereas the HLF uses six bulbs, which were focused on to the point coincident with the X-ray and sample. Both the QLF and HLF were capable of temperatures up to 2000°C, though the maximum temperature achieved in these experiments was ∼1600°C. Two optical furnaces and beamlines were used due to beamtime availability. In preparation for optical furnace XRD experiments, each powder sample was mixed with ∼5 wt% Pt which served as an internal reference material and as a heat conductor, ensuring even heating. Pt peaks in the XRD patterns were used to calculate the temperature of each scan (Touloukian, 1975 ▸). Sample and Pt powder samples were mounted in sapphire (Crytur Co., Czech Republic) or fused-silica capillaries (Charles Supper Company) at the end of an alumina tube. Each sample was held in the goniometer head of the diffractometer.

Debye–Scherrer (transmission) geometry powder XRD experiments were conducted with a flat panel area detector. The X-ray wavelength at 17 BM-B at APS, was 0.24117 Å (65.554 keV), with a sample-detector distance of ∼1002 mm which was refined with an external CeO_2_ (SRM 647b NIST standard). Data were collected between 1.5 and 12.5° 2θ (*d* ^−1^ 0.217–1.795 Å^−1^) with a step size of 0.001°. The X-ray wavelength at 28 ID-2 at BNL was 0.1847 Å (67.12 keV), with a sample-detector distance of ∼1423 mm at BNL, which was refined with an internal Pt reference material.

### 2.4. Data processing and analysis

Synchrotron area detector image data were azimuthally integrated with the program *GSAS-II* (Toby & Von Dreele, 2013 ▸). Changes in the crystal structures were determined using Rietveld refinement (Rietveld, 1969 ▸; Loopstra & Rietveld, 1969 ▸) applied to XRD data. This analysis was performed with Bruker *TOPAS* v.5 (Bruker, 2007 ▸; Coelho, 2018 ▸). Rietveld refinement parameters included phase scale, background, atom positions, atomic displacement factors, and occupancy for certain atoms. A more detailed Rietveld procedure is given in supporting information. The high-temperature crystal structure was solved using Fourier difference maps in *TOPAS* and changes in space group symmetry checked with *PLATON* (Spek, 2020 ▸, 2009 ▸). Structures and Fourier difference map iso-surfaces were plotted using *VESTA* (Momma & Izumi, 2008 ▸).

### 2.5. Thermal expansion calculation

The thermal expansion tensor, **α**, is a description of the three-dimensional crystallographic change as a function of temperature (Jones *et al.*, 2013 ▸; Paufler & Weber, 1999 ▸). It is a second rank tensor, the components of which, denoted as α_*x*_, are called the coefficients of thermal expansion. The linear and volumetric coefficients of thermal expansion are calculated from the temperature-dependent length or volume (at 25°C) as shown in equations (1) and (2) below.

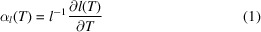




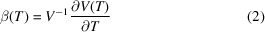




The unit-cell parameters as a function of temperature were fit to second-order polynomials with the least-squares method. These quadratic polynomials [*a*(*T*) and *c*(*T*)] were then used to calculate the axial, linear thermal expansion value [α_*a*_(*T*) and α_*c*_(*T*)] via equation (1). A discussion of the functional forms used to fit the unit-cell parameters for a thermal expansion calculation is included in the supporting information Section S2.3. The linear α value can be converted to volumetric β value through the following equation for the hexagonal symmetry: β = 2α_*a*_+ α_*c*_ (Taylor, 1998 ▸), where α_*a*_ and α_*c*_ describe the thermal expansion in the *a* and *c* unit-cell axis directions, respectively. The average linear thermal expansion value used here is β/3. Additional details describing α and its calculation can be found elsewhere (Taylor, 1998 ▸; Schlenker *et al.*, 1975 ▸; Paufler & Weber, 1999 ▸; Touloukian, 1975 ▸; Jessen & Küppers, 1991 ▸).

## 3. Results and discussion

This section presents the thermal evolution of the crystal structures and thermal expansion values for the CaZP, SrZP, NTP and NZP compositions. The CaZP and SrZP compositions also include a high-temperature structure solution and investigation into the thermal expansion mechanism. The thermal expansion of MgZP is also included in the supporting information. For each dataset, (i) tabulated unit-cell parameter values, (ii) a corresponding diffraction pattern file (.xye), (iii) *TOPAS* output file ( .out), and (iv) a crystallographic information format file (.cif) can be reached by the online repository at the hyperlink in the supporting information.

### 3.1. CaZr_4_P_6_O_24_ and SrZr_4_P_6_O_24_

CaZP and SrZP were studied, including the crystal structure solution of a high-temperature polymorph, phase transformation, thermal expansion values, and thermal expansion mechanism. The *in-situ* XRD data were collected at beamline 17 BM-B at the APS.

#### 3.1.1. Crystal structure solution

*In-situ* synchrotron data were collected from 25 to 1500°C. The diffraction patterns showed the disappearance of several peaks on heating to 1262 (±4)°C for SrZP and 1268 (±2)°C for CaZP, indicating a phase transformation from the low-temperature 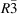 (space group No. 148) space group to a higher symmetry one. These peaks reappeared on cooling. Fourier difference mapping in *TOPAS* (Bruker, 2007 ▸; Coelho, 2018 ▸) was used to determine the locations of missing and excess electron density in the unit cell. Trial-and-error was involved in determining the correct choice of atoms to move to best describe the phase transition. Adding a 0.5 occupancy Sr (or Ca for CaZP) atom to the (0,0,0) site worked best to account for the electron density movement in the high-temperature structure. Additional information can be found in the supporting information Section S2.2.

**Figure 4:**
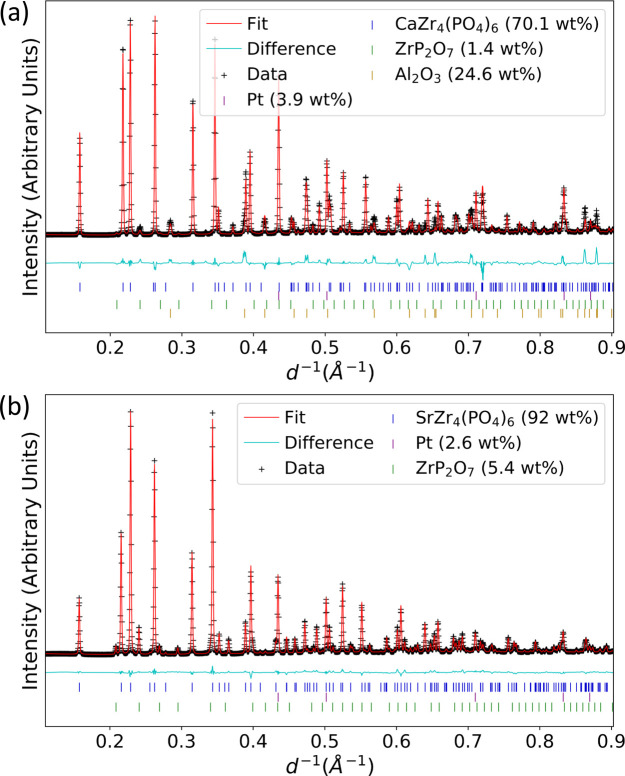
Synchrotron diffraction pattern and calculated diffraction patterns from the Rietveld refined structure for the new high-temperature polymorph of (*a*) CaZP at 1420 (±2)°C and (*b*) SrZP at 1409 (±4)°C. Pt is the internal reference material for temperature measurement and ZrP_2_O_7_ is a minor phase.

The ADDSYM function in *PLATON* (Spek, 2020 ▸, 2009 ▸) was used to determine changes in symmetry that could be present within a given crystal structure. It suggested that the new polymorph has 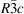 (space group No. 167) symmetry. Powder XRD patterns and Rietveld refined structure of the high-temperature polymorphs, refined with this new structure for CaZP and SrZP, are displayed in Fig. 4 ▸. Crystallographic and Rietveld data are provided in Table 1 ▸. The same high-temperature polymorphs were found in both CaZP and SrZP. Impurities are present in both specimens, including ZrP_2_O_7_ due to off-stoichiometry synthesis, and Pt which was added as a reference material as described in Section 2.3.

**Figure 5:**
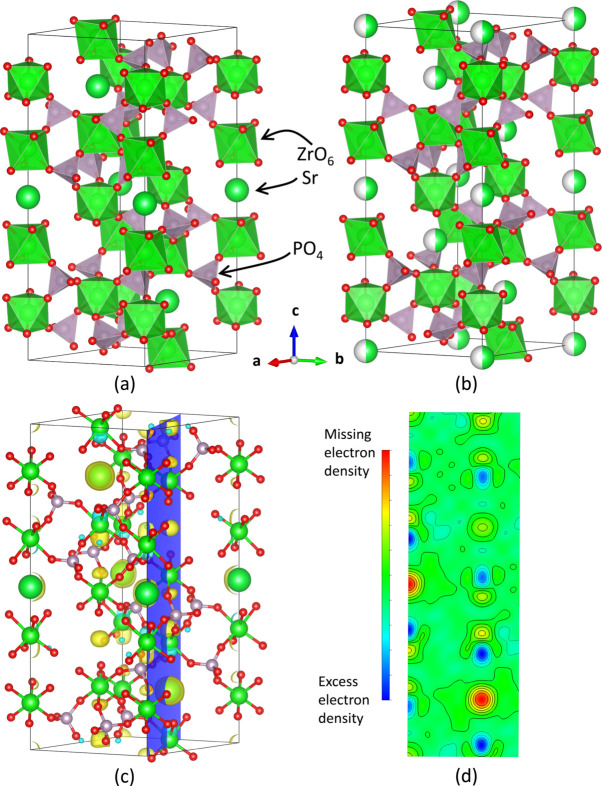
(*a*) Low-temperature, low-symmetry phase in 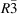 (space group No. 148), (*b*) high-temperature, high-symmetry phase in 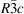 (space group No. 167), (*c*) iso-surface plot from the Fourier difference map showing differences in electron density from the low-temperature phase with missing electron density shown in yellow and excess electron density shown in light blue, and (*d*) cross section of the iso-surface plot to provide more detail in the variation in intensity.

The crystal structures of SrZP, including the low- and high-temperature polymorphs, are shown in Fig. 5 ▸ with parameters for the 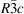 structure given in Table 1 ▸. Fig. 5 ▸ also shows how the Fourier difference map and misplaced electron density from the low-temperature structure was used to determine the location of missing Sr atoms within the unit cell at the (0, 0, 0) site (and the symmetry related locations) for the high-temperature polymorph. This shows that the Sr^2+^ cation goes from being ordered (only on the *M*1 sites) to being disordered (randomly distributed on the *M*1 and *M*2 sites). The solution method and resulting structure for the high-temperature CaZP polymorph are analogous to SrZP.

**Figure 6:**
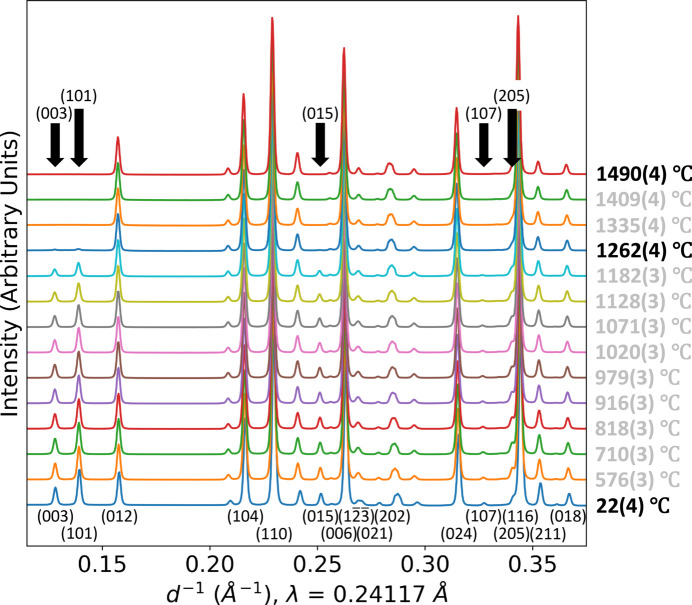
Temperature series on heating of SrZP XRD patterns showing the phase transformation to a higher symmetry space group. Peaks that are absent in the high-temperature polymorph are labeled with black arrows and their Miller plane indices.

The change in XRD patterns with temperature is shown in Fig. 6 ▸ as SrZP undergoes the phase transformation. This transition is reversible and was measured on heating and cooling (though it is only shown on heating here). The systematic absence of peaks in this pattern can also be used to determine the new space group. The phase transformation is associated with the loss of several peaks in the XRD pattern of the high-temperature phase, as shown in Fig. 6 ▸, including the peaks shown in Table 2 ▸. The systematic absence of certain peaks matched the reflection condition shown in Table 3 ▸ and corresponded to a *c* glide. This added symmetry came from the added Sr site at (0,0,0) with 0.5 occupancy found by Fourier difference mapping and the *PLATON* (Spek, 2020 ▸, 2009 ▸) search for symmetry. Using both the reflection conditions for the absent peaks and the Fourier difference maps were good self-consistency checks for this high-temperature crystal structure. A comparison with other CaZP studies is given in the supporting information section.

#### 3.1.2. Unit-cell data and coefficients of thermal expansion

It was shown previously (Woodcock *et al.*, 1999*a* ▸; Alamo, 1993 ▸) that the thermal expansions between related NZP-type materials in each of these 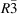 space groups tend to show positive thermal expansion in the *a* and *c* axis directions, whereas 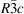 show negative thermal expansion in the *a *axis and positive expansion in the *c* axis. The phase transformation from 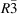 to 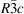 in the Ca_1–*x*_Sr_*x*_Zr_4_P_6_O_24_ solid solution presented an opportunity to understand the differences in *a* unit-cell axis expansion.

Additionally, CaZP and SrZP have markedly different thermal expansions within the 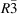 phase. The *a* unit-cell axis contracts on heating for CaZP, whereas it expands for SrZP. The *c* unit-cell axis expands for both. Negative thermal expansion in the *a* and *b* unit-cell axes in CaZP is perhaps unique among the NZP-type materials that crystallize in the 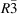 space group (Alamo, 1993 ▸). Most NZP-type materials in the 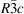 space group show negative thermal expansion in the *a* axis. The *a* axis changes from positive to negative thermal expansion at a composition of *x* = 0.45 in Ca_1–*x*_Sr_*x*_Zr_4_P_6_O_24_, as shown in supporting information.

This creates the opportunity to learn more about both the mechanism leading to the difference in anisotropy in the expansion of the *a* axis between Ca and Sr, as well as the changes in expansion through the phase transformation from 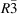 to 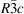 , while keeping cations on the other sites constant. Previous studies probing these differences in thermal expansion did so by examining different compositions of NZP-type materials with substitutions on the *M*1/*M*2 and *R* sites, which led to more possible differences between the materials (bond strength, cation size, electron orbitals) that could influence the thermal expansion.

**Figure 7:**
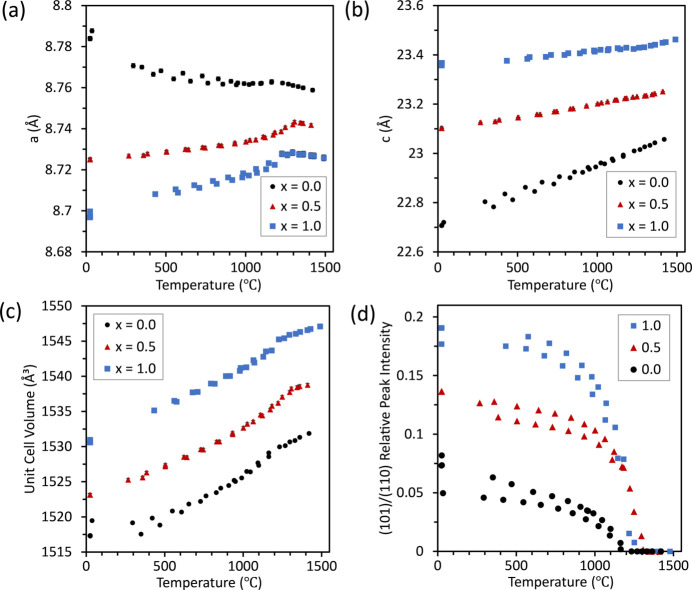
Temperature-dependent, unit-cell parameters (*a*) *a* and (*b*) *c*, (*c*) unit-cell volume, and (*d*) relative integrated peak intensity of (101)/(110) in Ca_1–*x*_Sr_*x*_Zr_4_P_6_O_24_, for compositions *x* = 0.0, 0.5, and 1.0. The transformation can be seen here in (*a*) by the changes in slopes of the *a* unit-cell parameters and in (*d*) by the decreases in the (101) peak intensities. Data were collected on both heating and cooling, leading to variability in values for each composition. Most error bars are smaller than the data markers.

All Ca_1–*x*_Sr_*x*_Zr_4_P_6_O_24_ samples were shown to be stable from 25 to 1500°C. The unit-cell parameters and volume for three representative samples are shown below in Fig. 7 ▸. The change in slope of the unit-cell parameters versus temperature shows the phase transformation temperature. Additional compositions in the Ca_1–*x*_Sr_*x*_Zr_4_P_6_O_24_ solid solution can be found in the supporting information.

**Figure 8:**
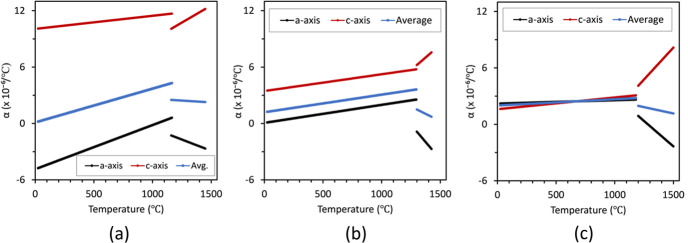
The temperature-dependent thermal expansion (α) for (*a*) CaZP, (*b*) Ca_0.5_Sr_0.5_Zr_4_P_6_O_24_ and (*c*) SrZP plotting the thermal expansion coefficients along the *a* axis, *c* axis, and average linear thermal expansions.

The *a* and *c* axes, and average thermal expansion values are summarized in Fig. 8 ▸ for both low- and high-temperature phases. In the low-temperature 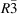 phase, the *a* unit-cell parameter underwent negative thermal expansion for Ca-rich samples in the Ca_1–*x*_Sr_*x*_Zr_4_P_6_O_24_ system up to *x* = 0.45, after which the *a* axis had a positive thermal expansion. The volumetric thermal expansion was also lowest for these samples from 25 up to ∼500°C, at which point the coefficient of thermal expansion continued to increase and at the upper range of temperatures it had the higher coefficient of thermal expansion. The thermal expansion was almost isotropic for SrZP, with nearly overlapping *a* axis and *c* axis values. The Ca-rich end of the solid solution experienced greater anisotropy in thermal expansion with a larger positive *c*-axis thermal expansion and a negative *a*-axis thermal expansion. This anisotropy between the *c* and *a* axes led to an overall very similar average thermal expansion throughout the CaZP–SrZP solid solution, with average values between −1 and 4 × 10^−6^ per °C.

In the high-temperature 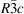 phase, unit-cell parameters behaved similarly for both CaZP and SrZP. The *a* unit-cell parameter experienced negative thermal expansion, whereas the *c* parameter underwent positive thermal expansion. The average linear thermal expansion had values from 0.7 × 10^−6^ per °C to 2.5 × 10^−6^ per °C with the lower values measured at higher temperatures. This type of a and *c* axis thermal expansion is typical of NZP-type materials in the 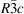 space group (Lenain *et al.*, 1987 ▸; Alamo, 1993 ▸).

Ca_0.5_Sr_0.5_Zr_4_P_6_O_24_ was previously found to have an average linear thermal expansion of 0°C^−1^ (Limaye *et al.*, 1991 ▸; Breval *et al.*, 2000 ▸), however that was not observed here. Only CaZP underwent a negative average linear thermal expansion, and only from 25 to 200°C (as seen in the supporting information for a specimen in which more data points were collected in the low-temperature range). At temperatures above 500°C it began to expand faster than the Sr-rich end of the solid solution Ca_1–*x*_Sr_*x*_Zr_4_P_6_O_24_.

All compositions in this solid solution had low average thermal expansion values of less than 4.5 × 10^−6^ per °C. The high-temperature phase also had low thermal expansion values, in fact the average thermal expansion value decreased for all compositions of Ca_1–*x*_Sr_*x*_Zr_4_P_6_O_24_ above the phase transformation temperature. SrZP had the lowest anisotropy in coefficients of thermal expansion between the *a* and *c* axis directions. While CaZP had a smaller thermal expansion value in the *a* axis than any other in this solid solution, it also had the largest thermal expansion along the *c* axis. This large anisotropy in CaZP would likely lead to increased strain in polycrystalline solids. Studies of dilatometry-derived thermal expansion values of bulk NZP-type materials have sometimes differed from XRD-derived crystallographic values, which has been attributed to densification when sintering as well as strain between grains due to anisotropic thermal expansion values along the *a*- and *c*-axis directions (Evans, 2002 ▸).

#### 3.1.3. Thermal expansion mechanism

**Figure 9:**
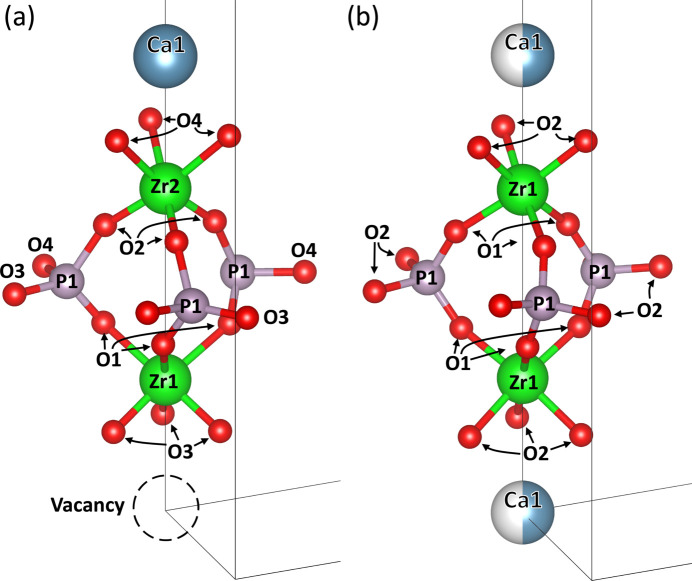
A selected subset of the CaZP unit cell illustrated with atom labels to depict the corner-sharing nature of the PO_4_ tetrahedra and ZrO_6_ octahedra for the (*a*) low temperature, 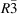 (space group No. 148), and (*b*) high temperature, 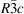 (space group No. 167). The Ca, Zr and vacancy in this subset lie along the *c*-unit cell axis. The same labeling scheme is used for SrZP.

Investigation of the underlying atomistic mechanism leading to the observed thermal expansion involves examining the changes in groupings of atoms such as interatomic distances and polyhedral angles. It is important to have the language to describe the different unique atomic sites within the unit cell. A subset of the CaZP unit cell with atom labels is shown in Fig. 9 ▸ for the 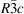 and 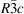 space groups; these atoms must be labeled separately because each space group generates different atomic positions/labels.

Previously, several methods have been used to investigate the thermal expansion behavior in low and negative thermal expansion materials, including interatomic distances, angles involving bridging oxygen atoms (*M*—O—*M* angles) (Tao & Sleight, 2003 ▸; Evans *et al.*, 1996 ▸) and the rotation of a polyhedron relative to the rest of the unit cell (Alamo, 1993 ▸). These methods are complementary and the benefits of using both have been demonstrated in related systems of materials (Woodcock *et al.*, 1999*a* ▸). Both methods were employed here to describe the underlying crystallographic changes that took place during the thermal expansion and phase transformation. There are several interatomic angles and distances that are related or describing part of the same interaction of atoms, so some covariance was expected.

**Figure 10:**
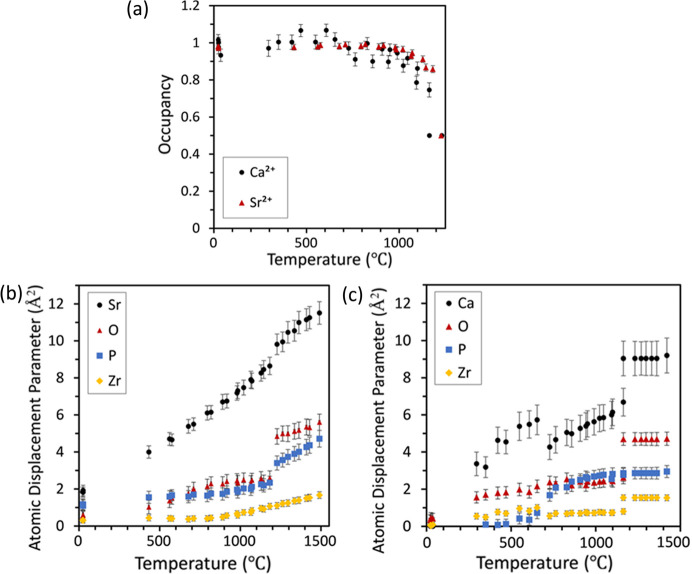
Occupancy (*a*) of the Ca and Sr cations shows significant disordering of the *M*1 and *M*2 sites before the phase transition temperature. The dependence of temperature on ADP for (*b*) SrZP and (*c*) CaZP. Data are shown for both heating and cooling of specimens.

*Interatomic distances, cation-bridging oxygen angles and atomic displacement parameters.* Fig. 10 ▸ tracks the atomic displacement parameters (ADPs), selected interatomic distances, and angles for both SrZP and CaZP. The ADPs in Figs. 10 ▸(*b*) and 10 ▸(*c*) increase with higher temperatures, as expected due to increased thermal motion. There is a discontinuity in the ADP at the transformation temperature, which is likely due to the way atoms are represented in each space group. While they have the same number of atoms per unit cell, the low-temperature crystal structure in 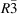 has twice as many atoms explicitly represented as does the structure in 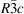 . The space group 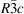 has more symmetry-generated atoms in the unit cell.

The *M*1 and *M*2 sites demonstrate some differences, relative to the atoms surrounding them, between SrZP and CaZP. The distance between Zr2–Sr1 of 3.52 Å is larger than the 3.34 Å between Zr2–Ca1. This also corresponds to a larger Zr–vacancy distance for SrZP than for CaZP. Zr–*M*1 cation and Zr–vacancy distances each converge just before the transformation in which there is disordering of Sr or Ca to *M*1 and *M*2 sites. There is a larger difference between the *M*1 site size (Zr2–Sr1) and the *M*2 vacancy size (Zr1–Vac) for SrZP than for CaZP.

**Figure 11:**
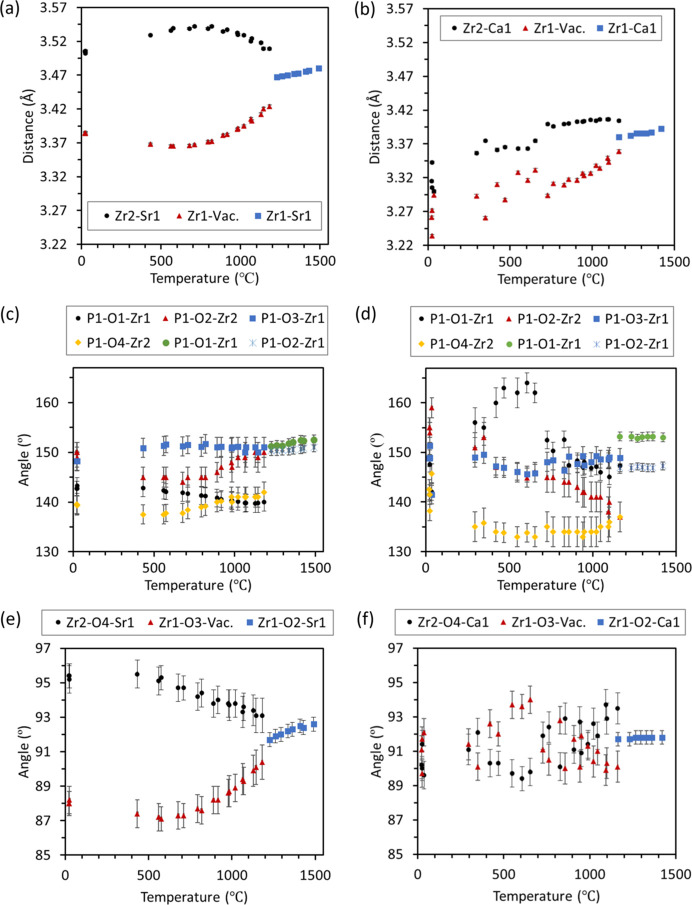
The distance from Zr1/Zr2 to the *M*2/*M*1 site is shown for (*a*) SrZP and (*b*) CaZP, illustrating the merging of the *M*1 and *M*2 sites. Cation-bridging oxygen (*M*—O—*M*) bond angles between both 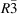 and 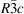 phases for P—O—Zr are presented for (*c*) SrZP and (*d*) CaZP, as well as the Zr—O—*M*1/*M*2 angles for (*e*) SrZP and (*f*) CaZP. Data are shown for both heating and cooling of specimens.

Cation-bridging oxygen angles (*M*—O—*M* angles) are illustrated in Fig. 11 ▸ for both SrZP and CaZP, including the P1—O—Zr in the 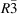 and 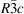 phases. In 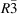 , the P—O*x*—Zr*x* angles in Figs. 11 ▸(*a*) and 11 ▸(*b*) indicate that the P1—O1—Zr1 angle appears to converge to the same value as P1—O4—Zr2, and P1—O2—Zr2 with P1—O3—Zr1 for SrZP just before the phase transformation temperature. This convergence to similar values of P1—O*x*—Zr*x* angles is not seen in CaZP. For CaZP, there are large fluctuations in these P1—O*x*—Zr*x* angles. It would be expected that an increase in P1—O1—Zr1 and P1—O2—Zr2 angles would correspond to an increase in the *c* axis, and an increase in P1—O3—Zr1 and P1—O4—Zr2 angles would correspond to an increase in the *a* axis. In 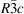 , P1—O1—Zr1 and P1—O2—Zr1 have similar values near 150° and each increase in value for SrZP. For CaZP in the 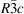 phase, the P1—O1—Zr1 and P1—O2—Zr1 are ∼152° and 148°, respectively, and stay approximately constant with temperature. While there are several differences in the behavior of these angles between CaZP and SrZP, it is hard to determine if these angles are useful in determining the differences in thermal expansion mechanism of between these materials.

The Zr—O—*M* angles in Fig. 11 ▸(*e*) experience a wide separation of 87 to 95°, then converging values near the phase transformation for SrZP. However, for CaZP these values are scattered near 91(±2)°C and do not show as clear a trend. This is consistent with the changes in the related bond distances given in Figs. 11 ▸(*a*),  ▸(*b*), S4(*c*) and S4(*d*), in which there are more clearly defined convergences of the Zr—*M* and *M*—O distances for SrZP than for CaZP. The difference seen between SrZP and CaZP appear to be due to the greater compression of the *M*2 site (vacancy) and expansion of the *M*1 site (Sr1) in SrZP, whereas the vacancy and Ca1 sites are more similar in size between the 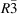 and 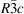 phases for CaZP.

*Polyhedral rotations.* Two types of polyhedral rotations were examined, one in the ZrO_6_ octahedra and another in the PO_4_ tetrahedra. This type of examination of NZP-type materials is like those of Alamo (Alamo, 1993 ▸; Alamo & Rodrigo, 1992 ▸) and Woodcock (Woodcock *et al.*, 1999*a* ▸, 1998 ▸). The rotation and distortion of each polyhedron was measured using their projections on to the *ab* plane, which helped to probe the atomistic mechanism leading to differences in expansion in the *a* and *b* axes between SrZP and CaZP. The mechanism of positive thermal expansion in the *c* axis has already been explained in terms of interatomic distances and angles.

**Figure 12:**
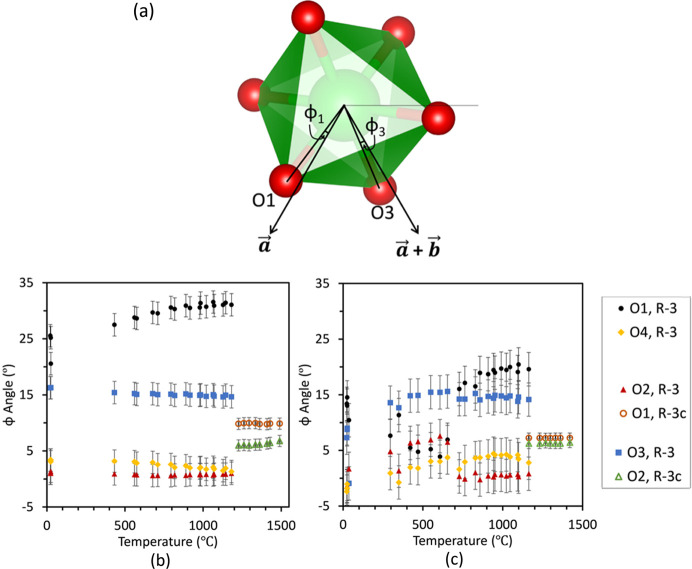
The φ angle is the angle between the Zr—O bonds and the *a* unit-cell axis (and the *a* + *b* direction) when the bond is projected onto the *ab* plane. Definition of the φ angle is depicted in (*a*) the 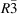 space group. Variation in the angle is systematically plotted for (*b*) SrZP and (*c*) CaZP. Datasets are shown for both heating and cooling.

The angle φ measures how O_1_, O_2_, O_3_ and O_4_ in a ZrO_6_ octahedron rotate about the *c* axis as illustrated and summarized in Fig. 12 ▸, where a ZrO_6_ octahedron is drawn with atom labels in both the 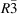 and 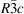 space groups. Fig S3 also depicts the definition of φ. The difference between φ values in the 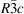 space group has also been used to measure the distortion between the top and bottom layer of atoms within an octahedron(Alamo, 1993 ▸; Woodcock *et al.*, 1999*a* ▸). There is a larger range of φ values for the SrZP phase from about 0 to 33°, compared to −2 to 20° for CaZP. For both phases, there is a larger difference between φ angles for SrZP than for CaZP, which corresponds to a greater distortion (differing rotations of the top and bottom oxygen layers) of the ZrO_6_ octahedron in SrZP. The largest rotations for both CaZP and SrZP occur in the φ_1_ (φ for the O1 atom) in both phases, which corresponds to the oxygen atom that is bonded between Zr1 and P1, which is farther from the occupied *M*1 site.

**Figure 13:**
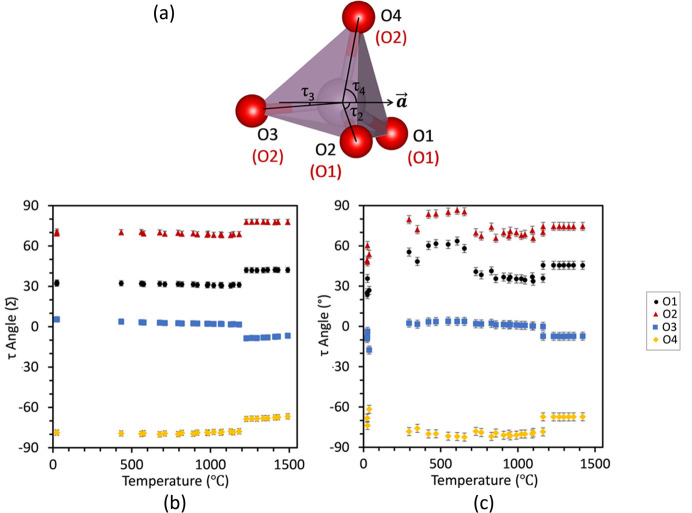
The (*a*) τ angle is the angle that the P1—O*x* bond direction makes with the *a* axis when projected on the *ab* plane is plotted for (*b*) SrZP and (*c*) CaZP. Definition of the τ angle is shown in (*a*) the 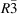 space group in black text and the 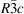 space group in red text. Thermal evolution of τ is described with the 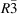 nomenclature. Datasets are shown for both heating and cooling.

The angle τ projects a PO_4_ tetrahedron on to the *ab* plane, then measures the angle of the P1—O*x* vector relative to the *a* axis, as summarized in Fig. 13 ▸. Unlike the φ-angle, which gives a measurement relative to Zr1/Zr2, which lie on the *c* axis, P1 does not reside on any unit-cell axis, so differing P1—O*x* values of τ cannot be compared directly. For both SrZP and CaZP, the τ angles have a discontinuity in their values at the transformation temperature from the 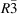 (low temperature) to 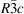 (high-temperature) phases. At the phase change, τ1, τ2, and τ4 all increase and τ3 decreases, describing a sudden rotation of PO_4_ tetrahedra relative to the *a* and *b* axes. The τ_1_ and τ_2_ values only for CaZP each increase by 20 to 30° between 300 and 700°C, but do not in SrZP, suggesting that a more distorted or rotated PO_6_ tetrahedron exists in this temperature range in CaZP. This temperature range also corresponds to the increase in distance of P1 from the *c* axis shown in Fig. 13 ▸(*b*). However, the uncertain and large error bars for both the φ and τ angles make comparison of the relatively minor changes in these angles difficult to determine differences that could cause significant changes in the thermal expansion between SrZP and CaZP.

There was a clear rotation of both ZrO_6_ and PO_4_ polyhedra during the 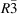 to 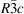 transformation, seen in both the CaZP and SrZP. In the 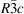 structures, the thermal expansion for both CaZP and SrZP was similar to other NZP-type materials when the *R* site is filled with Zr^4+^. While the *a* axis contracts in both the 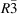 and 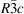 phases of CaZP, it is likely that they do so through different mechanisms because their *a* unit-cell parameters have different slopes (and different thermal expansion values). Both SrZP and CaZP show expansion along the *c* axis in 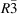 and 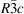 and contraction along the *a* axis in 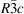 . The mechanism behind the opposite signs for these *a*-axis thermal expansions in the 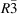 phase is still unclear. Previous studies have suggested that the reason could be due to opposite rotations of the polyhedra; however, that was not definitively seen here. There were several small differences between the rotations of ZrO_6_ octahedra and PO_4_ tetrahedra and small changes in *M*—O—*M* bonds, but there were no major differences in the direction of rotation for these polyhedra. What can be concluded, however, is that the *M*1/*M*2 site in SrZP went through larger variations in size than did the *M*1/*M*2 sites in CaZP, as measured by the *M*1/*M*2 to Zr distance. The vacancy on *M*2 in SrZP was larger than on the *M*2 site than CaZP and it went through larger changes in size than CaZP.

In the 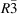 structures, no significant differences in value or rotation direction of the ZrO_6_ octahedra and PO_4_ tetrahedra between SrZP and CaZP were observed to explain the opposing thermal expansions along the *a* axis, in contrast to previous predictions. However, there is larger uncertainty in the angles and distance measurements that involve oxygen atoms than those only involving other atoms, which likely comes from the lower atomic scattering factor of oxygen than the other higher atomic number atoms in these materials (Henke *et al.*, 1993 ▸). It is still possible that PO_4_–ZrO_6_ rotations are the thermal expansion mechanism leading to the differences in anisotropy along the *a* axes in CaZP and SrZP; however, that cannot be conclusively shown from this powder XRD data. The *M*1 cation size affects the size and compressibility of the *M*2 site vacancy, which is likely important for the differences in thermal expansion. Future directions to investigate the positive and negative thermal expansions in the *a* axis of SrZP and CaZP include measurements that are sensitive to local changes in bonding, in addition to total scattering experiments to probe local order in addition to the average structure measured in diffraction experiments.

### 3.2. NaTi_2_(PO_4_)_3_ thermal expansion coefficients

NaTi_2_(PO_4_)_3_ **(**NTP) crystallizes in the 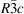 space group and maintains this crystal structure for the entire range studied from 25 to 1425°C. Above this temperature the specimen melted and/or reacted with the sapphire capillary. This extended the previous temperature range in a powder diffraction study by a few hundred degrees (Ribero *et al.*, 2016 ▸). This higher temperature range is of interest due to the change in *a*-axis thermal expansion and the opportunity to examine the difference.

**Figure 14:**
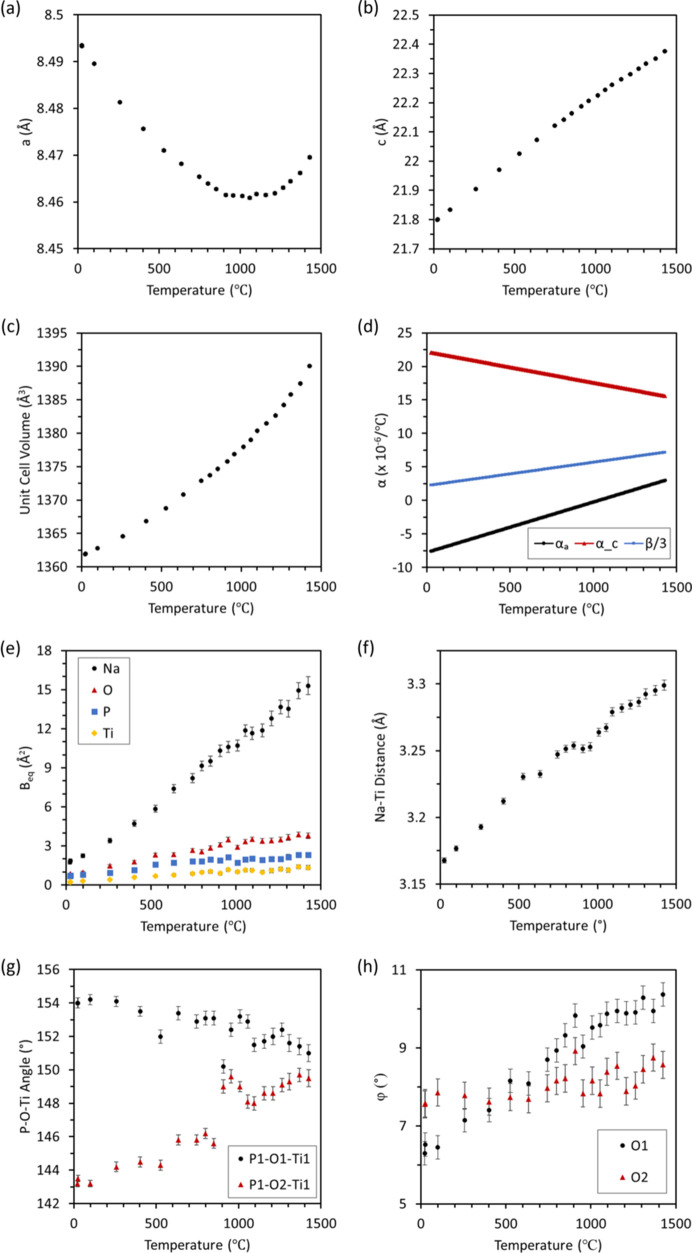
NaTi_2_(PO_4_)_3_ temperature dependent (*a*) *a* axis and (*b*) *c* axis unit-cell parameters, (*c*) unit-cell volume, (*d*) thermal expansion coefficients along the *a* and *c* axes, and average linear thermal expansion, (*e*) atomic displacement parameters, (*f*) Na^+^ to Ti^4+^ distance which describes the Na^+^ site size, (*g*) P—O—Ti inter-polyhedral angles, and (*h*) φ angles describing TiO_6_ octahedral rotation. Datasets are shown for both heating and cooling.

Unit-cell parameters and thermal expansion values are shown in Fig. 14 ▸. While there was no phase change in the measured temperature range and the *M*1 and *M*2 sites were each fully occupied by the Na^+^ cations, there was still a change from negative to positive thermal expansion of the *a* axis of the unit cell above 1100°C. This is uncommon in the NZP-type materials in space group 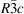 , as most show a contraction along the *a* axis for all temperatures (Alamo, 1993 ▸).

### 3.3. NaZr_2_(PO_4_)_3_ thermal expansion coefficients

**Figure 15:**
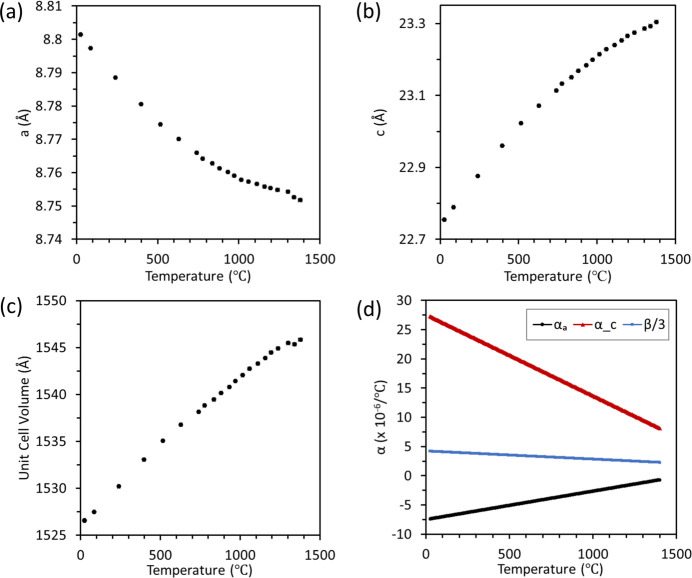
NaZr_2_(PO_4_)_3_ temperature dependent (*a*) *a* axis and (*b*) *c* axis unit-cell parameters, (*c*) unit-cell volume, and (*d*) thermal expansion coefficients along the *a* and *c* axes, and average linear thermal expansion. Datasets are shown for both heating and cooling measurements.

NaZr_2_(PO_4_)_3_ (NZP) crystallizes in space group 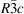 and maintains this crystal structure for the entire range studied here from 25 to 1378 (±10)°C. The average linear thermal expansion values varied from 4.2 × 10^−6^ per °C at 25°C to 2.3 × 10^−6^ per °C at 1380°C. It exhibited a highly anisotropic behavior between the negative thermal expansion in the *a* axis and positive one in the *c* axis as displayed in Fig. 15 ▸. Because there were no phase changes or significant variations in thermal expansion coefficients, the thermal expansion mechanism was not explored further here.

## 4. Conclusion

CaZP, SrZP, NaTP, and NZP powders were synthesized and their thermal expansion values, phase stability, and thermal expansion mechanisms measured from 25 to 1500°C via *in-situ*, synchrotron diffraction. A high-temperature polymorph of CaZP and SrZP was solved with Fourier difference mapping and Rietveld refinement, its crystal structure is in the 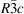 space group. This phase is present above 1268 (±2)°C and 1262 (±4)°C for CaZP and SrZrP, respectively. The new phase shows disorder on the *M*1 and *M*2 sites where it has 0.5 occupancy of the cation Ca^2+^ or Sr^2+^ on each site. The low-temperature 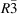 structure shows an ordering of the Ca^2+^ or Sr^2+^ on the *M*1 and *M*2 sites with an occupancy of 1 on the *M*1 site and 0 on the *M*2 site.

The linear thermal expansion of the Ca_1–*x*_Sr_*x*_Zr_4_P_6_O_24_ solid solution was measured as between −1 and 6 × 10^−6^ per °C in the 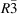 phase and between 0 and 3 × 10^−6^ per °C in the high-temperature 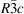 phase. The 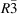 phase for both Ca and Sr showed negative thermal expansion of the *a* axis and positive thermal expansion of the *c* axis. CaZP showed the lowest thermal expansion from 25 to 200°C where it had a negative average thermal expansion value (between −1 and 0 × 10^−6^ per °C).

Negative and positive thermal expansions along the *a* axis of the 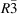 phase of CaZP and SrZP, respectively, was investigated. The *M*1 (Ca) and *M*2 (vacancy) sites in CaZP were more similar in size than in SrZP in which the vacancy was more compressed at low temperatures. This suggests that the compressibility of each cation site is important to the difference in thermal expansion. However, there was not a clear relation between the differences in *a*-axis thermal expansion and the inter-polyhedral angles or relative rotation of PO_4_ tetrahedra or ZrO_6_ octahedra, as was suggested previously (Woodcock *et al.*, 1998 ▸, 1999*b* ▸; Lenain *et al.*, 1987 ▸; Alamo, 1993 ▸; Alamo & Rodrigo, 1992 ▸). This implies the need for additional measurements. Neutron total scattering (Dove *et al.*, 2002 ▸) can combine long-range order, using Bragg peaks, with short-range order, derived from diffuse scattering. Total scattering experiments have been useful in examining rigid body rotations caused by low-frequency vibrational modes in other network structures (Tucker *et al.*, 2005 ▸; Baise *et al.*, 2018 ▸) and would be a good characterization technique to study the thermal expansion mechanism in SrZP and CaZP. Total scattering enabled the measurement of disordered structural materials in which local structural changes are different from the structural average described from diffraction experiments using Bragg peaks.

A combination of both X-ray pair distribution function (XPDF) and X-ray absorption fine structure (XAFS) led to a study of the changes in a network structure (Cao *et al.*, 2003 ▸; Bridges *et al.*, 2014 ▸) that were caused by low-energy vibrational modes. A recent study that combined temperature-dependent solid-state NMR, neutron diffraction, and computation DFT (Morgan *et al.*, 2022 ▸) examined Na_l+*x*_Zr_2_Si_*x*_P_3–*x*_O_12_. It could be useful to apply these techniques to understanding the differences in *a*-axis positive and negative expansions in the CaZP-SrZP system.

NaTP was shown to undergo a change in the thermal expansion of its *a* axis from negative to positive at 1100°C. Previously, only a negative thermal expansion in the *a* axis was reported. An investigation of the differences in the thermal expansion mechanism in the positive and negative expansion regions did not support changes in the rotation direction of the interconnected PO_4_ tetrahedra and TiO_6_ octahedra described previously. NaTP is another example illustrating that the trend that NZP-type materials in the 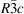 space group undergo negative thermal expansion in the *a* axis and positive thermal expansion in the *c* axis is not valid for all temperatures ranges or compositions.

## 5. Related literature

The following references are cited in the supporting information: Chakraborty *et al.* (2005 ▸), Fischer *et al.* (2004 ▸), Gregg *et al.* (2013 ▸), Liu *et al.* (2018 ▸), Alamo & Rodrigo (1992 ▸), Momma & Izumi (2008 ▸), Hill (1992 ▸), Hill & Cranswick (1994 ▸), Bruker (2007 ▸), Coelho (2018 ▸), McCusker *et al.* (1999 ▸), Dinnebier *et al.* (2018 ▸), Toby & Von Dreele (2013 ▸), Hulbert & Kriven (2023 ▸), Hulbert *et al.* (2021 ▸), Allen (1974 ▸), Quan (1988 ▸), Montgomery *et al.* (2012 ▸), Deshpande & Mudholker (1961 ▸), Jones *et al.* (2013 ▸, 2012 ▸), Jones (2012 ▸), Langreiter & Kahlenberg (2015 ▸), Kempter & Elliott (1959 ▸), Weber *et al.* (1998 ▸), McCormack *et al.* (2018 ▸), Seymour *et al.* (2016 ▸, 2015 ▸), Knight *et al.* (1999 ▸, 1996 ▸), Ballirano & Melis (2009 ▸), Saxena & Shen (1992 ▸), Küppers (2013 ▸), Cline (2020 ▸), Brown (1988 ▸), Dean-Mo & Brown (1992 ▸, 1993 ▸), Kabekkodu *et al.* (2002 ▸).

## Supplementary Material

Crystal structure: contains datablock(s) global, SrZr4PO46, phase_1_R-3c_SrZr4PO46, pxrd_JB1008A_14_1400_maskSapphire.xye, CaZr4PO46, phase_1_R-3c_CaZr4PO46, pxrd_JB1007A_16_1400_maskSapphire.xye. DOI: 10.1107/S2052520624001598/yh5031sup1.cif

Rietveld powder data: contains datablock(s) pxrd_JB1007A_16_1400_maskSapphire.xye. DOI: 10.1107/S2052520624001598/yh5031phase_1_R-3c_CaZr4PO46sup2.rtv

Rietveld powder data: contains datablock(s) pxrd_JB1008A_14_1400_maskSapphire.xye. DOI: 10.1107/S2052520624001598/yh5031phase_1_R-3c_SrZr4PO46sup3.rtv

Includes Figs S1-S11 and Tables S1- S16. DOI: 10.1107/S2052520624001598/yh5031sup4.pdf

Raw X-ray diffraction data (.XYE), TOPAS input (.INP) and output (.OUT) files for NZP materials, including SrZr4P6O24, CaZr4P6O24, NaTi2P3O12, and NaZr2P3O12: http://doi.org/10.5281/zenodo.8121948

CCDC references: 2333634, 2333635

## Figures and Tables

**Table 1:** Crystallographic and Rietveld data for the high-temperature polymorph of CaZP and SrZP with estimated standard uncertainties show in parenthesis

	CaZr_4_P_6_O_24_					SrZr_4_P_6_O_24_				
Crystal data										
Crystal family	Hexagonal					Hexagonal				
Space group (number)	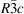 (167)					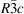 (167)				
*Z*	3					3				
*T* (°C)	1420 (2)					1409 (4)				
*a*, *b* (Å)	8.72614 (7)					8.76035 (8)				
*c* (Å)	23.4444 (4)					23.0338 (4)				
α, β (°)	90					90				
γ (°)	120					120				
Unit-cell volume (Å^3^)	1546.02 (3)					1530.87 (4)				
Rietveld refinement										
Computer program	*TOPAS* (v. 5)					*TOPAS* (v. 5)				
Weight % - Rietveld†	92.0					92.8				
*R*_wp_ (%)‡	5.20					6.26				
*R*_exp_ (%)‡	1.22					1.13				
*R*_p_ (%)‡	4.22					4.72				
GoF (%)‡	4.25					5.53				
*R*_Bragg_ ‡	2.45					2.61				
	Fractional coordinate					Fractional coordinate				
Atom site label	*x*	*y*	*z*	Occupancy	*B*_eq_ (Å^2^)	*x*	*y*	*z*	Occupancy	*B*_eq_ (Å^2^)
Ca1 or Sr1	0	0	0.5	0.5	11.01 (17)	0	0	0.5	0.5	11.6 (4)
Zr1	0	0	0.14803 (5)	1	2.14 (5)	0	0	0.14685 (6)	1	1.97 (6)
P1	0.2873 (4)	0	0.25	1	2.99 (11)	0.2884 (4)	0	0.25	1	2.99 (12)
O1	0.0376 (7)	0.8282 (6)	0.6985 (2)	1	4.64 (12)	0.0297 (7)	0.8257 (6)	0.6960 (2)	1	5.36 (14)
O2	0.2000 (5)	0.1733 (6)	0.0939 (2)	1	4.64 (12)	0.1923 (6)	0.1680 (6)	0.0909 (2)	1	5.36 (14)

† Remaining wt% from Pt or minor phases.

‡ Values are as defined in Bruker *TOPAS* software.

**Table 2:** Tracking the loss of XRD peaks in Fig. 6 ▸ through their Miller–Bravais indices

Hexagonal Miller index	Hexagonal Miller–Bravais index	Peak appearance in high-temperature phase
003	0003	Absent
101	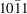	Absent
012	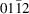	Present
104	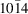	Present
110	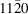	Present
015	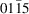	Absent
006	0006	Present
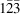	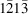	Present
021	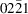	Present
202	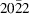	Present
024	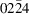	Present
107	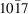	Absent
205	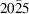	Absent
106	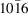	Present

**Table 3:** Systematic absence conditions† for hexagonal crystallographic coordinate systems

Type of reflections (Miller–Bravais indices)	Reflection condition	Glide vector	Symbol	Orientation of the plane
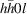	*l* ≠ 2	**c**/2	c	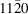
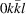				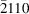
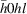				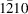

† From Table 2.1.3.4 in the *Teaching Edition* of *International Tables for Crystallography* (2021 ▸).
