# Supplementary material for: Crystal structure solution and high-temperature thermal expansion in NaZr2(PO4)3-type materials
Source: Acta Crystallogr B Struct Sci Cryst Eng Mater. 2024 Mar 22;80(Pt 2):146–59. doi: 10.1107/S2052520624001598 (PMC10994170; doi:10.1107/S2052520624001598)
Supplement: Supplementary file 4 [file b-80-00146-sup4.pdf]

STRUCTURAL SCIENCE
CRYSTAL ENGINEERING
MATERIALS

Volume 80 (2024)

Supporting information for article:

Crystal structure solution and high temperature thermal expansion in NaZr2(PO4)3-type materials

Benjamin S. Hulbert, Julia E. Brodecki and Waltraud M. Kriven

Supplemental material and tabulated data for the manuscript ‘Crystal structure solution and high temperature thermal expansions of $NaZr_2(PO_4)_3$-type materials.’ For each dataset in this study there is a corresponding diffraction pattern file (.xye), TOPAS output file (.out), and a crystallographic information format file (.cif) found in an online repository at the following hyperlink:

http://doi.org/10.5281/zenodo.8121948

S1. Additional Background Information

This was not the first temperature-dependent diffraction study of the $Ca_{1-x}Sr_xZr_4P_6O_{24}$ solid solution (Chakraborty *et al.*, 2005; Fischer *et al.*, 2004), however it was the only structural study that reached temperatures high enough to measure the phase transformation. A thermal conductivity and heat capacity study (Gregg *et al.*, 2013) measured properties of CaZP and BaZP ($BaZr_4P_6O_{24}$) up to 1800 ℃, but did not measure the $R\bar{3}$ to $R\bar{3}c$ phase transformation in CaZP (neither by in-situ XRD up to 1350 ℃ nor by DSC/TGA), but they were studied in an argon atmosphere. However, they did measure a similar phase transformation near 850 ℃ in BaZP. This could be because there is a smaller change in heat capacity associated with the phase change in CaZP or that the instrument is less sensitive/uncalibrated at the higher temperature at which the phase change in CaZP occurs versus BaZP. A $R\bar{3}$ to $R\bar{3}c$ phase transformation was measured in $NaSr_{0.5}Zr_4P_6O_{24}$ (Liu *et al.*, 2018) was different due to the partial occupancy differences between the Na^+ and Sr^{2+} cations. Another NZP-type material study (Alamo & Rodrigo, 1992) found a similar phase transformation in $NaSn_2(PO_4)_3$, except that the symmetry change from $R\bar{3}$ to $R\bar{3}c$ was due to the PO_4 tetrahedra, instead of the occupancy of the M1 and M2 sites.

The crystal structure of $Na_{l+x}Zr_2Si_xP_{3-x}O_{12}$, where x= 0 and 3 are shown in Fig. S.1 (a) and (b), along with three other NZP-type materials with other M-site atoms and occupancies. The different Na-sites (denoted M sites for a general cation at this location) can be compared between each material.

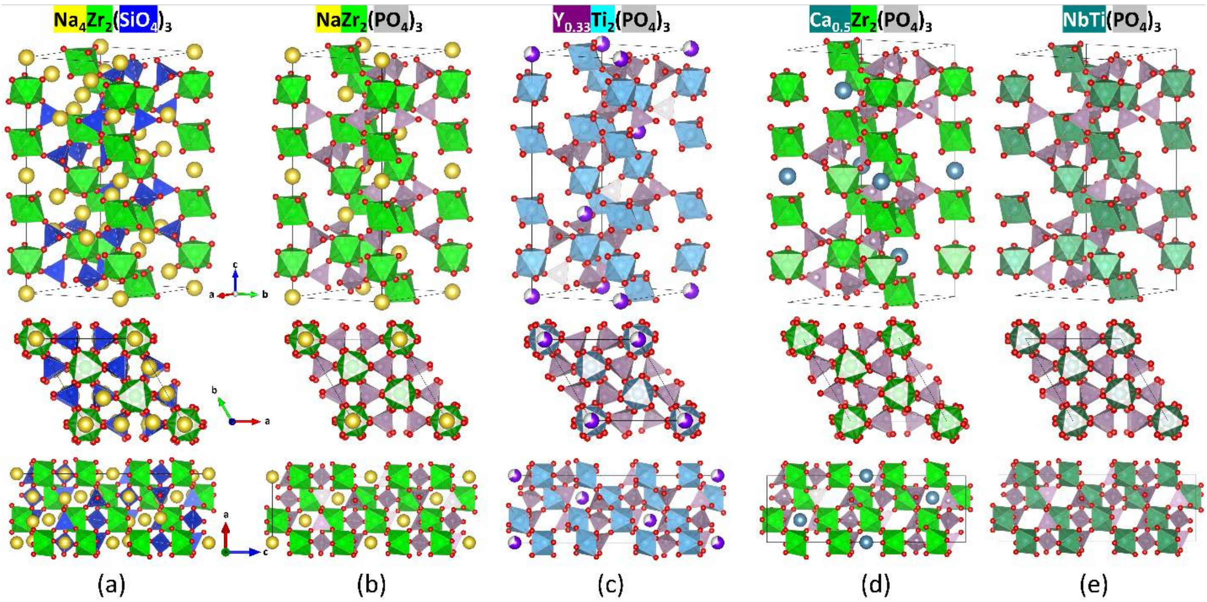

Figure S1 Crystal structures for (a) $Na_4Zr_2(SiO_4)_3$ (or $2Na_2O•2ZrO_2•3SiO_2$), (b) $NaZr_2(PO_4)_3$ (or $Na_2O•4ZrO_2•3P_2O_5$), (c) $Y_{0.33}Ti_2(PO_4)_3$, (d) $Ca_{0.5}Zr_2(PO_4)_3$, and (e) $NbTi(PO_4)_3$, showing the corner sharing nature ZrO_6 octahedra and PO_4 tetrahedra, with alternate views along the c-axis and b-axis. The additional locations and occupancies of the Na-sites (denoted M sites for a general cation at this location) can be compared between compositions. Orientations are shown from the same perspective using the program VESTA (Momma & Izumi, 2008).

S2. Rietveld Refinement and Thermal Expansion Calculations

S2.1. Rietveld Refinement Procedure

Due to the variability in results derived from Rietveld refined structures (Hill, 1992; Hill & Cranswick, 1994), the parameters and the order in which they were refined with TOPAS (Bruker, 2007; Coelho, 2018) version 5 are listed below. Descriptions of Rietveld refinement procedures and best practices can be found elsewhere (McCusker *et al.*, 1999; Robert E. Dinnebier *et al.*, 2018; Toby & Von Dreele, 2013). After each step below, two refinements were run.

1. Set minimum 2θ, and maximum 2θ, calculation step to 0.005°, primary goniometer radius to 9999 mm, secondary radius to 1000 mm, Lorentz Polarization factor fix at 90. Refine the scale of major phases and background (5th to 9th order Chebyshev polynomial as appropriate). Refine the specimen displacement distance (Hulbert & Kriven, 2023) at 25 ℃, then keep it constant for high temperature scans.
2. Add refinement of unit-cell parameters for major phases.

3. Set/fix the refined value for background (if refined with minor phases, it can cause the minor phases to account for some or most of the background). Add refinement of minor phase scale
4. Add refinement of minor phase minor phase unit-cell parameters. The result of this refinement is the starting point for the subsequent sample refinement.
5. Add refinement of B_{eq} to all atoms, fixing multiple sites for the same element to equal values. Atom labels are as shown in Fig. 9 and Table 1.

S2.2. Fourier Difference Map

The following lines of code were added to an input file to create the Fourier difference map via Launch mode in TOPAS. Mg atoms were added because they have roughly half the electron density of Sr. There is no option for a half occupancy Sr atom for the Fourier difference map section of TOPAS. FCF_Vesta is a useful function (https://topas.awh.durham.ac.uk/doku.php?id=fcf_vesta) that can be added to the local.inc file to create .fcf files that can be displayed in the program Vesta. The TOPAS (Bruker, 2007; Coelho, 2018) tutorials and user manual provide instructions describing instructions for Fourier difference maps.

```
fourier_map 1
load f_atom_type f_atom_quantity
    {
    Mg  = 6;
    }
fourier_map_formula = Fobs - Fcalc;
min_grid_spacing .2
FCF_Vesta(filename.fcf)
```

S2.3. Thermal Expansion Calculation

The following quotation comes from the supporting information of (Hulbert *et al.*, 2021):

> “Many functional forms have been used to describe lattice parameters or d_{hkl} values in a measured temperature interval during the calculation of α_{ij} and are given in Table S[1]. This is not an exhaustive list but shows the range of functions used in literature. The importance of selecting the appropriate functional form is apparent by the wide variety seen in literature and the need to calculate an accurate α_{ij}, yet a selection criterion has been absent from past literature. Here the choice of functional form chosen is determined quantitatively. The often-used coefficient of determination, R^2, does not provide a useful metric for choosing the

optimal polynomial order. For a data set with a limited number of points (as is often the case for synchrotron experiments), a larger R^2 does not necessarily lead to a better choice of functional form as it can lead to overfitting. Too high an order polynomial leads to an overfit set of data, which gives a good description of a specific dataset, but poor prediction of the future data points in the same range. To prevent overfitting, the Predicted Residual Sum of Squares (PRESS) statistic was calculated to determine the functional form of each lattice vector (Allen, 1974; Quan, 1988). This involved omitting each data point successively, determining a least-squares fit of all other data, then calculating the distance of the omitted data point from the fit function evaluated at that temperature, shown in **Eq. S1** (Montgomery *et al.*, 2012). The functional form with the lowest PRESS value was used to calculate a regression model for the lattice vectors, then calculate α_{ij}. In order to better compare α_{ij} between samples, the functional form with the lowest PRESS statistic for the majority of lattice parameters was used for all lattice parameters. This removed differences in how the α_{ij} values are calculated to allow comparison of data more easily. Some of the limitations of using a continuous function to describe lattice parameters in CTE calculations have been reported (Deshpande & Mudholker, 1961)."

$$P=\sum_{i=1}^{n}\left(y_i-\hat{y}_i(i)\right)^2 \qquad \text{(S1)}$$

Eq. S1. Calculation of the PRESS statistic (P) is a measure of the validity of a regression model. Each set of values (x_i, y_i) in the set of data ($i=1,\ldots,n$) is successively removed and a fit equation calculated ($\hat{y}_i$). The difference between the actual value, y_i, and the predicted value, $\hat{y}_i(i)$, is used to calculate the predicted residual sum of squares, P.

Table S1 Functional forms used to model lattice parameters or d_{hkl} spacing.

Function	Reference	Description
$l(T)=A_0 a+A_1 T$	(Jones *et al.*, 2013; Jones, 2012; Langreiter & Kahlenberg, 2015)	1st order polynomial
$l(T)=A_0+A_1 T+A_2 T^2$	(Kempter & Elliott, 1959; Weber *et al.*, 1998; Jones, 2012; Langreiter & Kahlenberg, 2015; McCormack *et al.*, 2018)	2nd order polynomial
$l(T)=A_0+A_1 T+A_2 T^2+$	(Weber *et al.*, 1998; Jones, 2012; Langreiter & Kahlenberg, 2015; McCormack *et al.*, 2018; Seymour *et al.*, 2016)	3rd order polynomial

$l(T)=A_0+A_1T+A_2T^2+$	(Jones *et al.*, 2013; Jones, 2012; Langreiter & Kahlenberg, 2015; Seymour *et al.*, 2016, 2015)	4th order polynomial
$l(T)=A_0+\frac{A_1}{(e^{A_2T}-1)}$	(Knight *et al.*, 1999; Knight, 1996; Ballirano & Melis, 2009)	Einstein equation, used for low temperatures
$l(T)=A_0+A_1T+A_2T^2+$	(Saxena & Shen, 1992; Küppers, 2013)	Used for high temperature minerals

l is a length, T is temperature, A_0, A_1, A_2, A_3, A_4, and A_5 are all constants

S3. Additional $Ca_{1-x}Sr_xZr_4P_6O_{24}$ Data

S3.1. Data Collection Details

Additional details for the datasets described in Table 1 are shown below in Table S2.

Table S2 Data Collection Information

Radiation Type	X-ray
Source, Laboratory	APS bending magnet, ANL
Beamline	17 BM-B
λ (Å)	0.241170
Sample-to-detector Distance (mm)	1001.8
2θ values (°)	$2\theta_{min}$ = 1.50, $2\theta_{max}$ = 12.50, $2\theta_{step}$ = 0.001
d^{-1}_{min}-d^{-1}_{max} (Å^{-1})	0.217 - 1.795
Atmosphere	Air
Specimen Container	Sapphire capillary
Material for Area Detector Image Calibration and Integration	NIST SRM 674b CeO_2 (Cline, 2020)
Heat Source	Quadrupole Lamp Furnace

S3.2. Unit-cell Data

The temperature dependent unit-cell parameters, volume, and $R\bar{3}$ (101) relative peak intensity data are shown in Fig. S2 for $Ca_{1-x}Sr_xZr_4P_6O_{24}$ where x=0.0, 0.2, 0.4, 0.5, 0.6, 0.8, and 1.0. The unit-cell parameters show a monotonic decrease for *a* and increase for *c* unit-cell parameters as a function of composition suggesting that only the M1 site is involved in incorporating the Ca^{2+} and Sr^{2+} cations.

Non-monotonic changes in unit-cell parameter as a function of composition were shown in other NZP-type solid solution systems in which the end members have their divalent cations residing on differing M1/M2 sites and occupancies (Liu *et al.*, 2018).

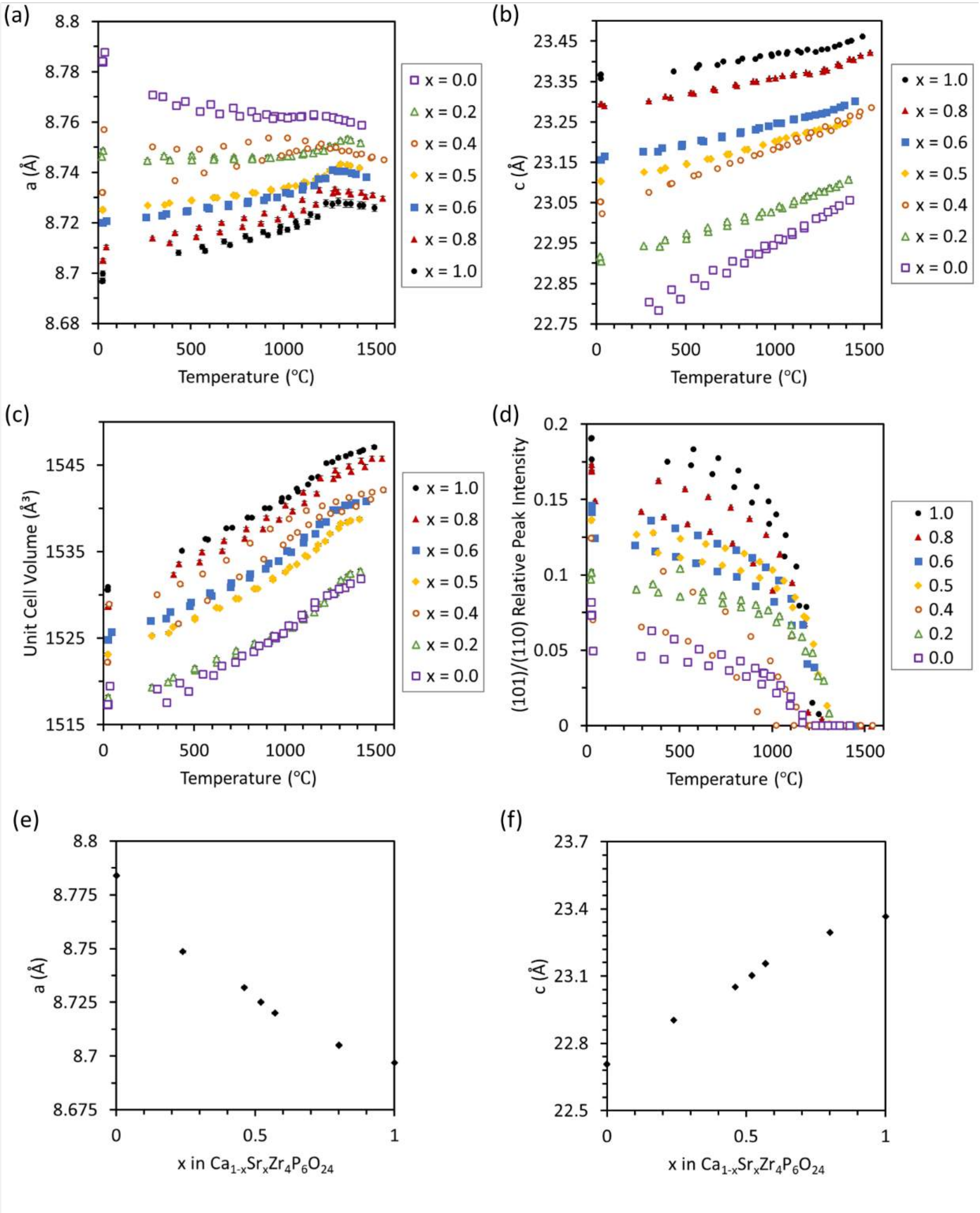

Figure S2 The (a) *a*, (b) *c* unit-cell parameters, (c) molar volume and (d) integrated intensity of the (101)/(110) peaks for the solid solution $Ca_{1-x}Sr_xZr_4P_6O_{24}$, where x=0.0, 0.2, 0.4, 0.5, 0.6, 0.8, and 1.0. The phase transition, measured by the disappearance of the 101 peak, occurs between 1150 and 1325 ℃ for all compositions, without a clear trend based on composition. The unit-cell parameters (e) *a* and (f) *c* as a function of solid solution composition x in $Ca_{1-x}Sr_xZr_4P_6O_{24}$ at 25 ℃. Each composition

includes values from both heating and cooling measurements. Most error bars are smaller than the data markers.

S3.3. Additional CaZP and SrZP Atom-Atom distances and bond angles

Figure S3 shows the definition of the φ angle, the angle between the Zr-O and the *a* axis or the *a* + *b* direction when projected onto the ab plane from a different perspective than was shown in Fig. 12.

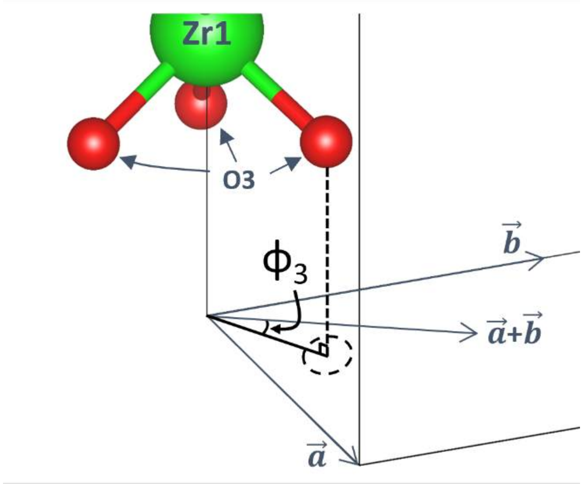

Figure S3 The φ angle is the angle between the Zr-O3 bond and the *a* + *b* direction when the bond is projected on to the ab plane.

The relocation of the M1-site cation at (0,0,0.5) (Ca^{2+} and Sr^{2+}, here) to the vacant M2 site (0,0,0) started at a lower temperature for CaZP than for SrZP. This is seen in the onset of the decrease in (101) peak integrated intensity in Fig. 7 (d) and the Rietveld refined occupancy in Fig. S4 (a). This movement is not surprising for the smaller and lighter Ca^{2+}. Once there is 0.5 occupancy on each M1 and M2 site, is what creates a *c*-glide plane perpendicular to both the *a* and *b* unit-cell axes and causes the systematic absence of several Bragg peaks, including the most intense (101) peak.

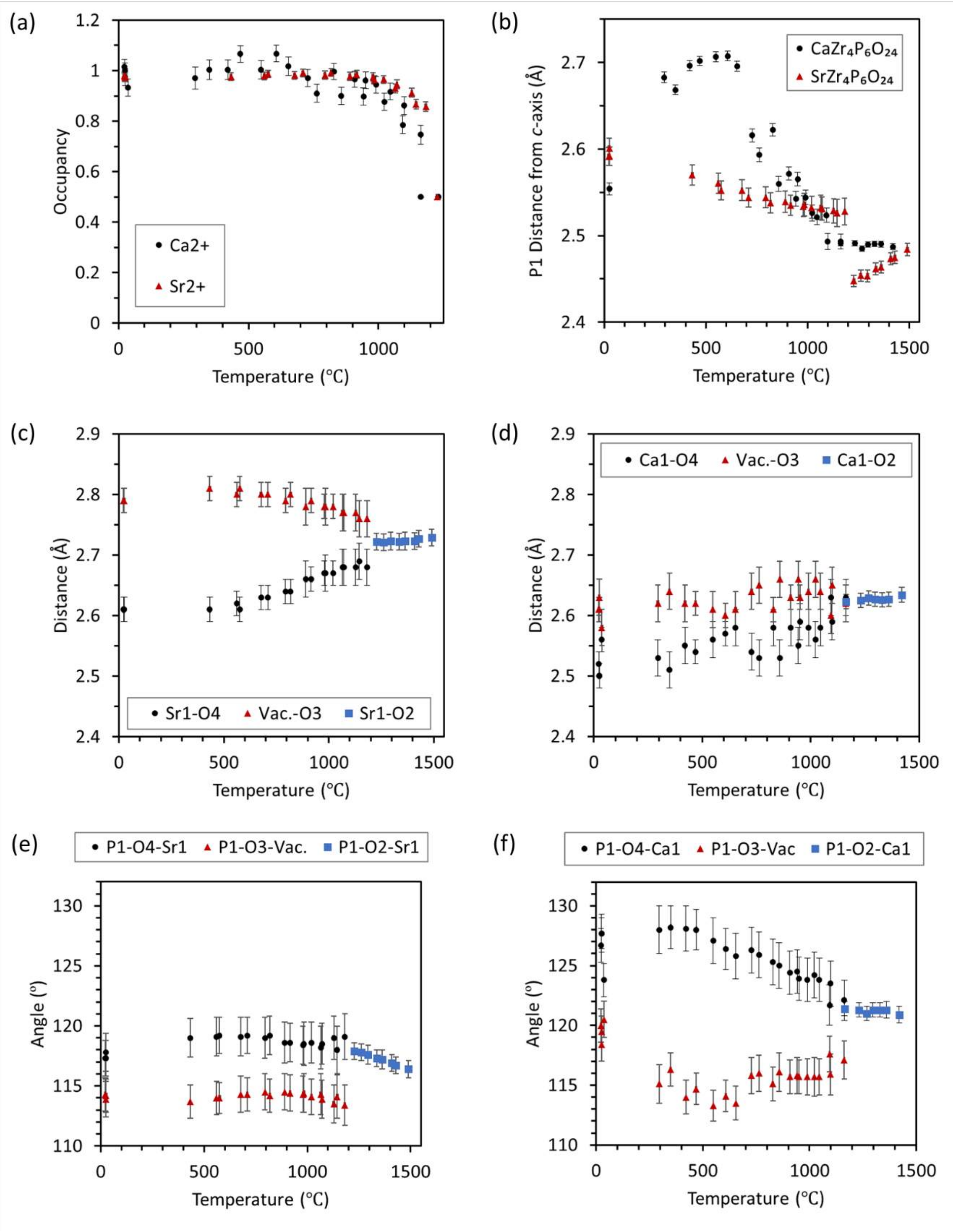

Figure S4 (a) The occupancy of Ca or Sr on the M1 site. (b) The distance from the P1 atom to *c* unit-cell axis. The distance from the M1/M2 site to the nearby O4/O3 site for (c) SrZP and (d) CaZP. These show the merging of several different sites in the high temperature polymorph. Cation-bridging oxygen (M-O-M) bond angles between both $R\bar{3}$ and $R\bar{3}c$ phases for P-O-M1/M2 for (e) SrZP and (f) CaZP. Values are shown both on heating and cooling.

The Sr1/Ca1-O4 distances are smaller than the vacancy-O3 distances, which shows the opposite effect from Sr1/Ca1-Zr2 and vacancy-Zr1. This is unsurprising, as the oxygen cation is initially more strongly attracted to the fully occupied M1 site before the phase transformation than it is to a vacancy. This effect is likely more pronounced for SrZP than for CaZP due to the larger vacancy in which the oxygen ions can move. These oxygens O3 and O4 are also bonded to Zr and P at a shared vertex, so it is plausible that these differences in vacancy size and Zr distance from the M1 site would cause the angle of connected PO_4 tetrahedron to be at a different distance or angle for SrZP and CaZP.

Fig. S4 (b) shows that P1 is farther from the *c*-axis up to about 750 ℃ for CaZP than it is for SrZP. This is somewhat unexpected because the CaZP a-axis has negative thermal expansion, whereas the a-axis in SrZP is positive. However, the movement of this PO_4 tetrahedron is also seen in the following sections in which various temperature dependent bridging oxygen angles and polyhedral rotations are examined.

In $R\bar{3}$ phase, there is a decrease in the P1-O4-Ca1 angle for CaZP from 128° to 121°, whereas the corresponding angle in SrZP stays approximately constant between 118° to 119°, in Figs. 11 (e) and (f). This shows that the O4 vertex of the PO_4 tetrahedra rotates relative to Ca1 in CaZP, but does not in SrZP.

S3.4. X-ray Fluorescence of the $Ca_{1-x}Sr_xZr_4P_6O_{24}$ Solid Solution

In order to determine the Ca to Sr ratio in the $Ca_{1-x}Sr_xZr_4P_6O_{24}$ solid solution accurately, the Rietveld refined ratio was compared with the XRF-determined value. This ratio is presented in Table S3, and it shows relatively good agreement between the two methods.

Table S3 Measured Ca^{2+} to Sr^{2+} Ratio in $Ca_{1-x}Sr_xZr_4P_6O_{24}$ by Rietveld refinement and X-ray Fluorescence

$Ca_{1-x}Sr_xZr_4P_6O_{24}$ label	x=0.0	x=0.2	x=0.4	x=0.5	x=0.6	x=0.8	x=1.0
Rietveld refined value	0.00	0.24	0.46	0.52	0.57	0.80	1.00
XRF determined value	0.00	0.22	0.44	0.53	0.63	0.82	1.00

S3.5. Differential Thermal Analysis of Phase Transition

In order to determine a phase transformation temperature more accurately than in XRD experiments (which was within about 100 ℃ for the experiments here), Differential Thermal Analysis (DTA) measurements were collected. A Shimadzu Q50 DTA instrument with two pans, one for the specimen with known mass, and one for the reference to measure the difference in heat flux as the specimen is heated. Measurements were collected at heating rates of both 20 ℃/min and 50 ℃/min. A slower rate has better precision (distinguishing nearby peaks), whereas a faster rate has higher sensitivity (for small heat flux differences) (Brown, 1988). A representative DTA dataset for 50 ℃/min heating rate is shown in Fig. S5, in which the phase transition near 1260 ℃ cannot be identified. The instrument was not sensitive enough to measure the order-disorder phase transition in $Ca_xSr_{1-x}Zr_4P_6O_{24}$ going from $R\bar{3}$ to $R\bar{3}c$. Future work could include high temperature Differential Scanning Calorimetry (DSC) measurements, because a well-calibrated DSC should have better sensitivity than the DTA (Brown, 1988). Because the M1 and M2 sites have become considerably disordered over a few hundred-degree range before the transition temperature, as shown in Fig. 7 (e), the signal associated with the phase transition will be relatively weak.

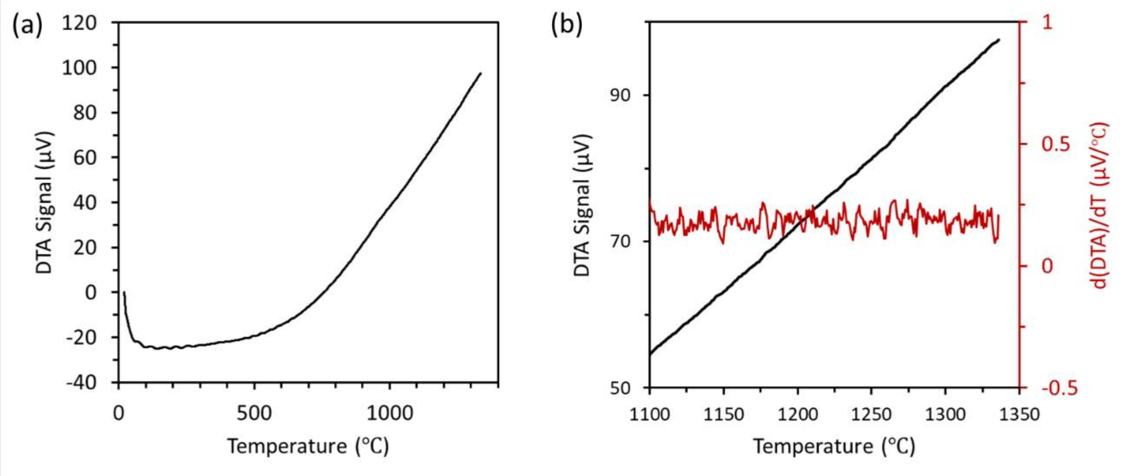

Figure S5 Differential Thermal Analysis measurement of $Ca_{0.5}Sr_{0.5}Zr_4P_6O_{24}$ heating at 50 ℃/min in which the phase transformation was unable to be measured showing (a) the full scan from 25 to 1350 ℃ and (b) a magnified subset showing the first derivative.

S3.6. Additional Dataset for CaZP

This CaZP dataset has higher resolution in the low temperature range is shown in **Fig. S6**. This shows that below 200 the average thermal expansion value is negative. The was not apparent in the dataset that reached higher temperatures, shown in the main body of the manuscript, because it only collected one datapoint in these temperatures.

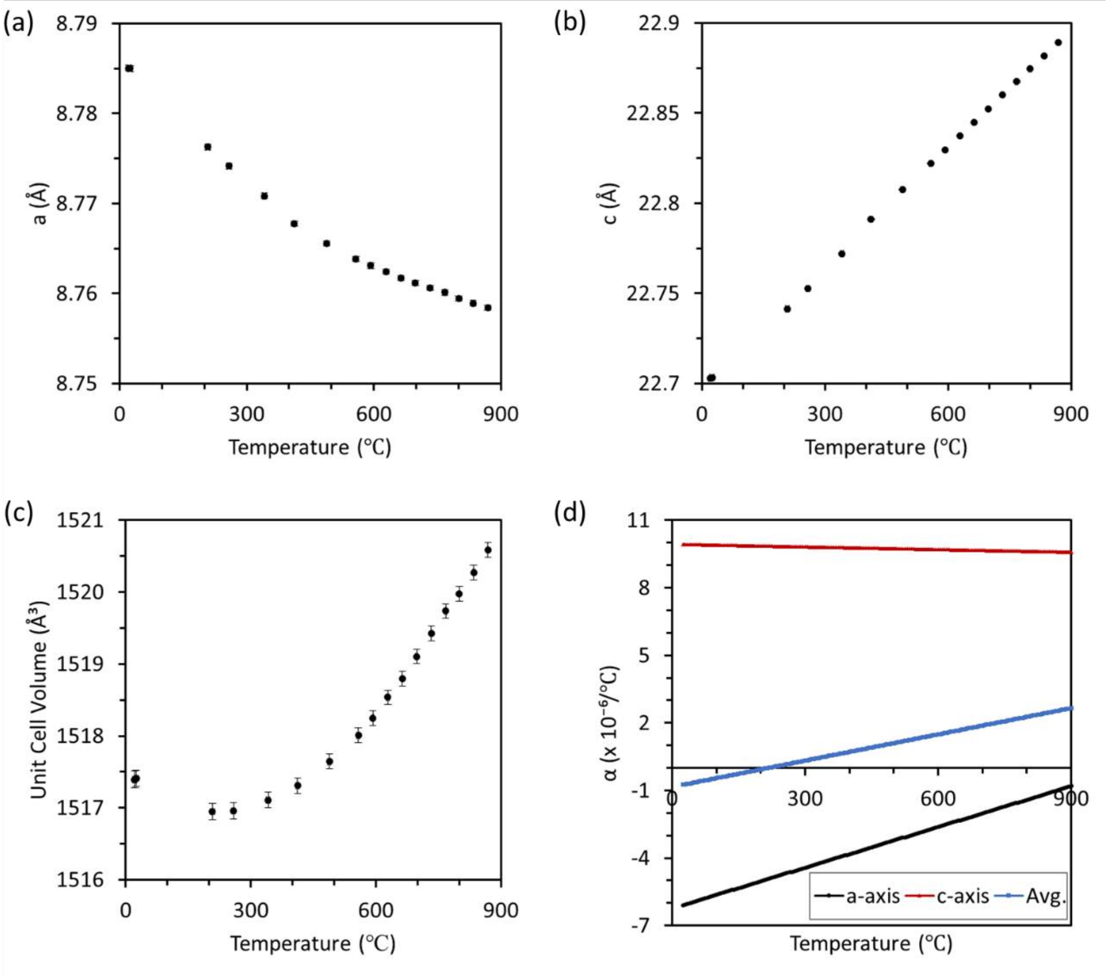

Figure S6 The (a) *a*, (b) *c* unit-cell parameters, (c) unit-cell volume and (d) thermal expansion coefficients for the solid solution CaZP for a dataset with more XRD patterns collected between 0 and 400 °C. The average linear coefficient of thermal expansion is negative below 200 °C, which was not evident in the dataset that probed a higher temperature range.

S3.7. $Ca_{0.55}Sr_{0.45}Zr_4P_6O_{24}$ Specimen

There is a composition at x = ~0.45 (x=0.44 as determined by Rietveld refinement from the 25 °C XRD scan, and x=0.46 as determined by XRF) at which there is a change in the anisotropy of the a and c-axis thermal expansion shown in Fig. S.7. This is the composition at which the a-axis goes from

having positive thermal expansion to having negative thermal expansion; x<0.45 shows negative thermal expansion in the a-axis and x>0.45 shows positive thermal expansion in the a-axis. It is unclear if the change in a-axis expansion seen here is kinetically driven hysteresis or due to the specimen beginning to decompose. The cooling rate during the XRD measurement in the QLF optical furnace (~100 ℃/min) is much faster than during the sample synthesis in a box furnace (2 to 10 ℃/min). No minor phases were found on cooling via XRD, however the maximum temperature reached for this specimen was 1543 (±7) C, which is near the decomposition temperature into CaO and ZrP_2O_7 that Gregg et al. (Gregg *et al.*, 2013) measured by high temperature DSC. It is possible that the amount of Sr in the specimen was slightly lower on cooling leading to the observed change in anisotropy. Regardless of which reason caused the change in a-axis expansion, it's clear that x=0.45 is very near the Ca to Sr ratio at which the a-axis of the unit cell goes from having negative to positive expansion in the $R\bar{3}$ phase.

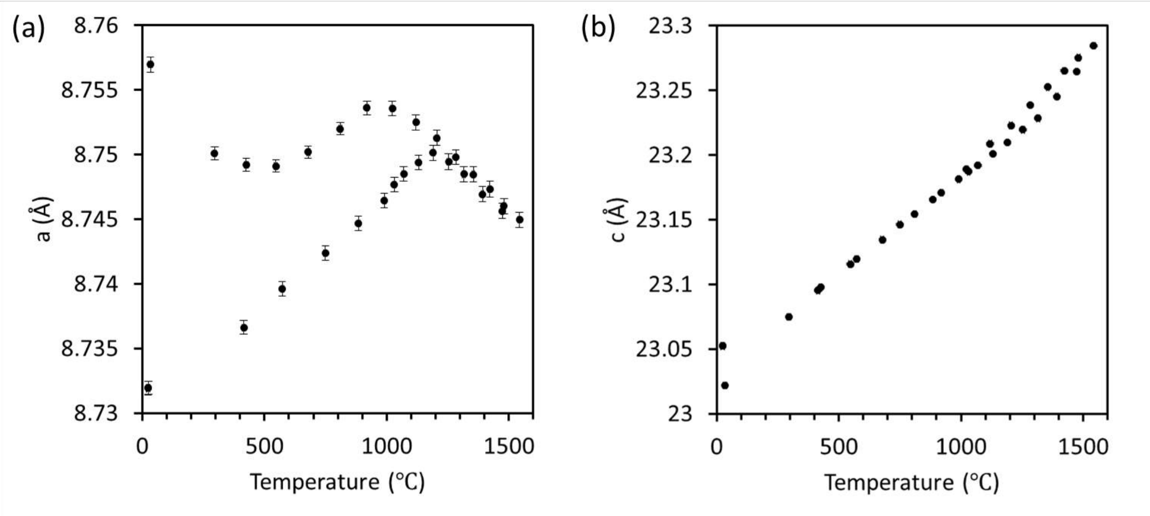

Figure S7 Temperature dependent unit-cell parameters for $Ca_{0.55}Sr_{0.45}Zr_4P_6O_{24}$ showing a change in anisotropy in the *a* unit-cell parameter on heating vs cooling. The unit-cell parameter is larger on cooling than on heating. Datasets are shown both on heating and cooling.

S3.8. Unit-cell Parameter Field map

The unit-cell parameter field map, in Fig. S8, compares the magnitudes of *c* and *a* unit-cell axes. There is a larger hysteresis in the unit-cell parameters on heating and cooling in **Figs. S2** and **S7** in the low temperature $R\bar{3}$ phase than in the high temperature $R\bar{3}c$ phase where there is no hysteresis seen. This could be because the framework structure of these samples is more rigid in the $R\bar{3}c$ phase than in the $R\bar{3}$ phase. An increased rigidity is likely due to having partial occupancy on both the M1 and M2 sites in the $R\bar{3}c$ phase, rather than the M2 site fully vacant in $R\bar{3}$. When there is a vacancy on

the M2 site, the surrounding PO_4 tetrahedra and ZrO_6 octahedra have greater room to rotate and move to accommodate changes in the unit cell.

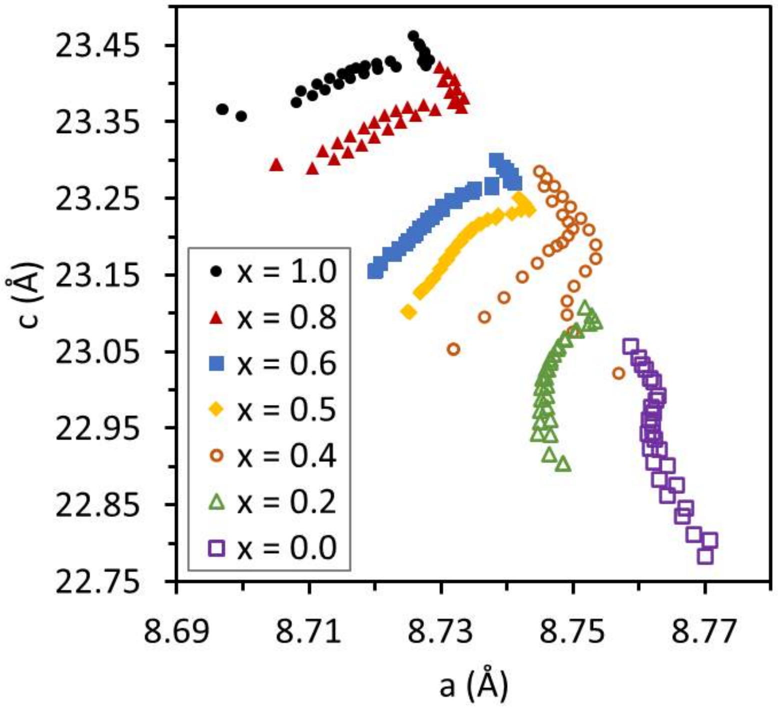

Figure S8 Field map of *c* vs *a* unit-cell lengths for $Ca_{1-x}Sr_xZr_4P_6O_{24}$ in both the $R\bar{3}$ phase than for the $R\bar{3}c$ phase. The high temperature $R\bar{3}c$ phase occurs at larger c-axis values and always have a negative slope. The a-axis shows greater flexibility in the structure for the $R\bar{3}$ phase in which there are a range of slopes than for the $R\bar{3}c$ phase. Datasets are shown for both heating and cooling.

S4. $MgZr_4P_6O_{24}$ Thermal Expansion Coefficients

$MgZr_4P_6O_{24}$ (MgZP) crystallizes in the $P2_1/n$ space group and maintains this crystal structure for the entire range studied here from 25 to 865 (±5) °C. The in-situ XRD data were collected at beamline 17 BM-B at the APS. Temperature dependent unit-cell parameters and calculated thermal expansion shown in Fig. S9. For the thermal expansion calculation, the unit-cell parameter *a* was fitted with a fourth order polynomial, b with a second order polynomial, c with a third order polynomial, and β with a fourth order polynomial. The average linear thermal expansion values varied from 2.3 $x10^{-6}$/°C at 505 °C to 5.7 $x10^{-6}$/°C at 865 °C. It had anisotropic thermal expansion values between each axis, with the lowest anisotropy seen near 600 °C. Because there were no phase changes or variations from the expected thermal expansion, the thermal expansion mechanism is not explored further here.

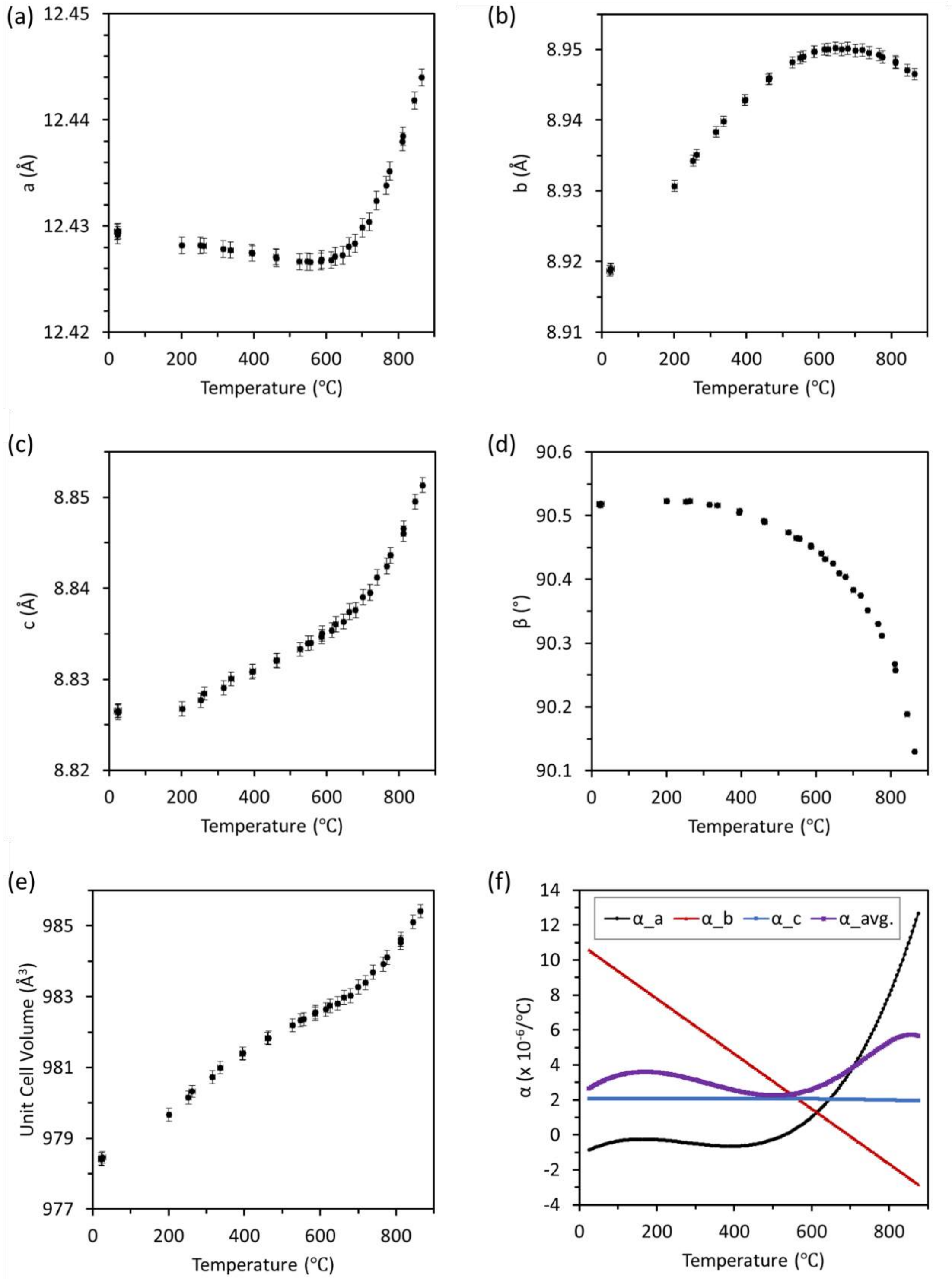

Figure S9 MgZP temperature dependent (a) *a*-axis, (b) *b*-axis, and (c) *c*-axis unit-cell parameters, (d) β angle, (e) unit-cell volume, and (f) thermal expansion coefficients along the *a*, *b*, and *c* axis directions, and average linear thermal expansion. Datasets are shown for both heating and cooling.

S4.1. $Mg_{0.5}Ca_{0.5}Zr_4P_6O_{24}$ Thermal Expansion and Unsolved Phase Transformation

$Mg_{0.5}Ca_{0.5}Zr_4P_6O_{24}$ was synthesized, but due to an error was made off stoichiometry and was not corrected before the synchrotron research trip due to time constraints. The resulting material has an unknown cation ratio and a secondary ZrP_2O_7 phase. The XRD pattern was similar to the $R\bar{3}$ CaZP phase, except with a few extra small peaks, as shown in Fig. S10. The thermal expansion, shown in Fig. S11, showed a change in slope when these peaks disappeared, suggesting that this is a new phase. This unknown phase had a phase transformation on heating between 390 and 450 °C to $R\bar{3}$. This transformation was reversible and transformed back to the unknown phase. Attempts to index it were unsuccessful due to the additional overlapping phases present (Pt reference material and ZrP_2O_7). To solve the structure of this new phase, it should first be made as a single phase, so its stoichiometry is known.

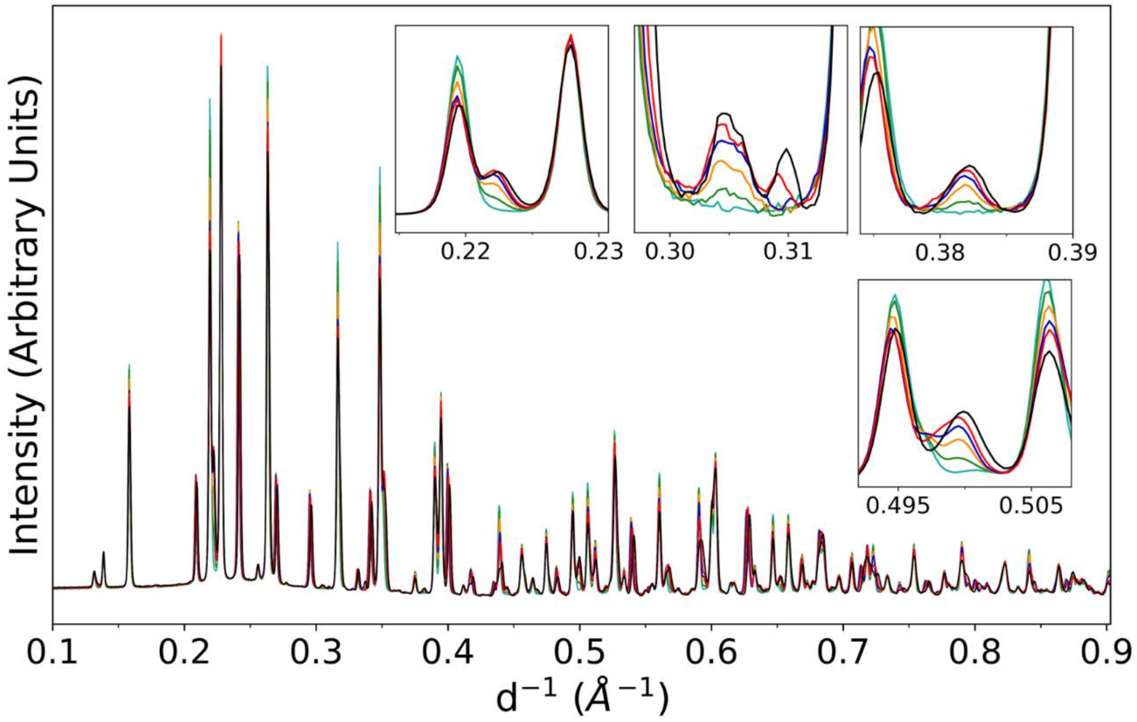

Figure S10 $Mg_{0.5}Ca_{0.5}Zr_4P_6O_{24}$ temperature dependent XRD patterns for temperatures from 25 (top, black line) to 448 (±8) °C (bottom, turquoise line) highlighting five Bragg peaks in the low temperature phase that are absent in the $R\bar{3}$ phase.

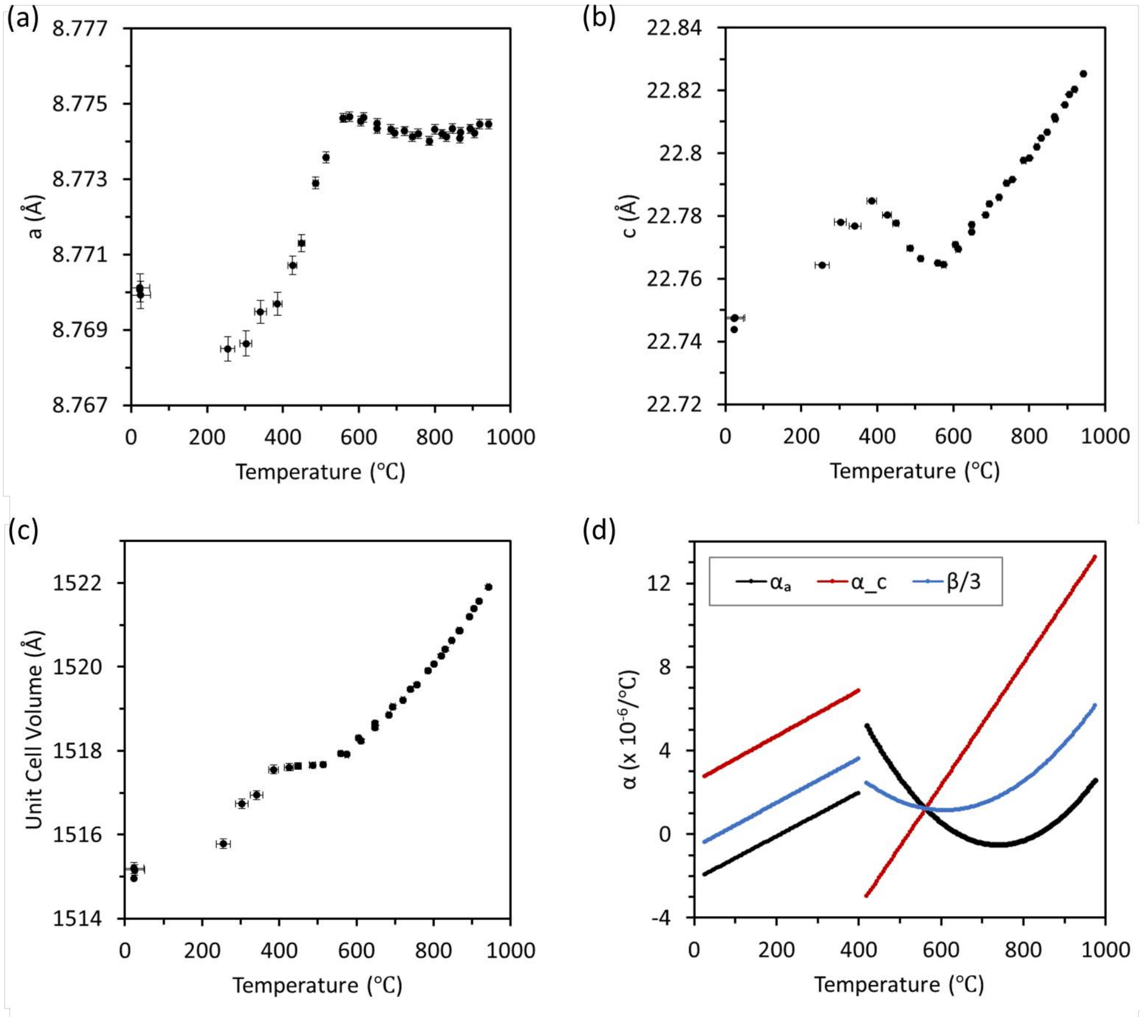

Figure S11 $Mg_{0.5}Ca_{0.5}Zr_4P_6O_{24}$ temperature dependent (a) *a*-axis and (b) *c*-axis unit-cell parameters, (c) unit-cell volume, and (d) thermal expansion coefficients along the a and c axes, and average linear thermal expansion. The unknown low temperature phase was refined in the $R\overline{3}$ space group. Datasets are shown for both heating and cooling.

The $Mg_{0.5}Ca_{0.5}Zr_4P_6O_{24}$ composition merits additional study. The solid solution $Mg_{1-x}Ca_xZr_4P_6O_{24}$ likely has an unsolved crystal structure which could be explored. Previous studies (Dean-Mo & Brown, 1992, 1993) on a part of this solid solution only covers the portion of the solid solution near CaZP in which the crystal structure stays in the $R\overline{3}$ space group. The (off-stoichiometry) $Mg_{0.5}Ca_{0.5}Zr_4P_6O_{24}$ sample synthesized here suggests that there is a new structure that exists between the end members of this solid solution. The change in symmetry between MgZP ($P2_1/n$) and CaZP ($R\overline{3}$) from incorporating the smaller Mg^{2+} cation into the structure comes from the ZrO_6 octahedra tilting so they are no longer parallel with the c-axis and a movement of the Mg^{2+} cation. This new phase might be due to a minor tilting of the ZrO_6 octahedra.

S5. Tabulated Data

Table S4 NZP-type materials in the hexagonal setting with substitutions on the M and R sites and used to create Fig. 2, gathered from the ICDD-PDF 4+ database (ICDD v 2022, International Center for Diffraction data, Newton Square, PA) (Kabekkodu *et al.*, 2002).

Composition	a (Å)	c (Å)	Space Group	PDF Card #
Ba Zr4 (P O4)6	8.6468	23.9436	R-3	04-015-1792
Ca Zr4 (P O4)6	8.7761	22.655	R-3	04-014-6377
Ca Zr4 (P O4)6	8.80703	22.7374	R-3	04-018-7054
Ca0.5 Zr2(PO4)3	8.7761	22.655	R-3	00-056-0637
Sr Zr4 (P O4)6	8.6937	23.299	R-3	04-014-6380
Sr0.5 Zr2 (P O4)	8.6937	23.299	R-3	00-056-0636
Sr1.5 Zr6 (P O4)	8.709253	23.33713	R-3	01-086-9977
La0.33 Zr2(PO4)3	8.738	23.216	R-3c	04-013-4168
K Zr2 (P O4)3	8.726	23.967	R-3c	00-035-0756
K Zr2 (P O4)3	8.71	23.89	R-3c	01-070-1905
Li Zr2 (P O4)3	8.847	22.24	R-3	01-070-6734
Rb Zr2 (P O4)3	8.71	24.3	R-3c	04-005-5501
Na Zr2 (P O4)3	8.792	22.842	R-3c	00-033-1312
Na Ge2 (P O4)3	8.146	21.504	R-3c	04-002-2741
Na Hf2 (P O4)3	8.755	22.746	R-3c	04-002-2743
Na Sn2 P3 O12	8.5087	22.5551	R-3c	00-049-1198
Na Ti2 (P O4)3	8.4854	21.7994	R-3	01-072-2457
Na Ge2 (P O4)3	8.105	21.52	R-3c	04-005-6030
Na Ge2 (P O4)3	8.098	21.53	R-3	04-010-5455
'Na Ti2 (P O4)3	8.486	21.802	R-3c	00-033-1296
'Ba Ti4 (P O4)6	8.348	23.01	R-3c	00-034-0094
'Ag Ti2 (P O4)3	8.475	22.106	R-3c	00-035-0737
'Li Ti2 (P O4)3	8.51	20.857	R-3c	00-035-0754
'K Hf2 (P O4)3'	8.6819	23.862	R-3c	00-038-1471
'Ca Ti4 P6 O24'	8.3754	22.0617	R-3	00-049-0787
'Li Hf2 (P O4)3	8.8306	22.027	R-3c	00-052-0569
'Li Ti2 (P O4)3	8.51183	20.84637	R-3cH	00-066-0871
'Na Ge2 (P O4)3	8.109	21.536	R-3	01-077-9936
'K Ti2 (P O4)3'	8.367	23.074	R-3c	01-079-1880
'Li Ge2 (P O4)3	8.275	20.47	R-3c	01-080-1922
'Mg0.5 Ti2 (P O4	8.4981	20.9746	R-3c	01-082-3833
'Na Ti2 (P O4)3	8.4854	21.7994	R-3c	01-083-7012
'Fe0.5 Ti2 (P O4	8.511	20.985	R-3	01-084-3899

'Li Sn2 (P O4)3	8.42	22.26	R-3c	04-001-7785
'K Hf2 (P O4)3'	8.694	23.765	R-3c	04-002-0331
'Na Hf2 (P O4)3	8.779	22.757	R-3c	04-002-0332
'Rb Hf2 (P O4)3	8.62	24.359	R-3c	04-002-0333
'Cs Hf2 (P O4)3	8.561	24.778	R-3c	04-002-0334
'Na Hf2 (P O4)3	8.755	22.746	R-3c	04-002-2743
'Sr Ti4 (P O4)6	8.3	22.62	R-3	04-002-6467
'Rb Ti2 (P O4)3	8.35	23.43	R-3c	04-005-5480
Ba0.5 Hf2(PO4)3	8.58	23.76	R-3c	04-005-5483
Sr0.5 Hf2(PO4)3	8.63	23.24	R-3c	04-005-5484
Ca0.5Hf2(PO4)3	8.82	22.65	R-3c	04-005-5485
Mg0.5Hf2(PO4)3	8.89	22.12	R-3c	04-005-5486
'Ba0.5 Ti2 (P O4	8.37	24.09	R-3c	04-005-5491
'Ca0.5 Ti2 (P O4	8.43	22.09	R-3c	04-005-5493
'Cs Ti2 (P O4)3	8.31	23.86	R-3c	04-005-5505
'K Ge2 (P O4)3'	7.996	22.522	R-3c	04-005-6028
'K Ti2 (P O4)3'	8.36	23.12	R-3c	04-006-5414
'Ti Nb (P O4)3'	8.56	21.92	R-3	04-006-9180
Eu0.33 Zr2(PO4)3	8.767	22.91	R-3c	04-007-6786
Pr0.33 Zr2(PO4)3	8.754	23.08	R-3c	04-007-6788
Nd0.33 Zr2(PO4)3	8.762	23.04	R-3c	04-007-6789
Sm0.33 Zr2(PO4)3	8.77	22.95	R-3c	04-007-6790
Gd0.33 Zr2(PO4)3	8.778	22.82	R-3c	04-007-6791
Tb0.33 Zr2(PO4)3	8.78	22.82	R-3c	04-007-6792
Er0.33 Zr2(PO4)3	8.796	22.64	R-3c	04-007-6793
Tm0.33Zr2(PO4)3	8.809	22.52	R-3c	04-007-6794
Yb0.33 Zr2(PO4)3	8.808	22.48	R-3c	04-007-6795
'Rb Ti2 (P O4)3	8.2896	23.53	R-3c	04-007-9051
'Cu Ti2 (P O4)3	8.531	21.336	R-3c	04-007-9675
'Cu Hf2 (P O4)3	8.84	22.128	R-3c	04-007-9676
'Ag Ge2 (P O4)3	8.066	21.819	R-3c	04-007-9677
'Ag Hf2 (P O4)3	8.76	22.794	R-3c	04-007-9681
'Ti Nb (P O4)3'	8.558	21.977	R-3c	04-009-4992
'Na Ti2 (P O4)3	8.4854	21.7994	R-3c	04-009-8561
'K Ge2 (P O4)3'	8.0066	22.566	R-3	04-010-6593
Cu0.5Ti (PO4)3	8.46	21.88	R-3	04-013-2049
La0.33Zr2(PO4)3	8.738	23.216	R-3	04-013-4168

Table S5 $Ca_{1-x}Sr_xZr_4P_6O_{24}$, x = 0.0 unit-cell parameter data

JB1007A_APS_17-BM-B_2020: Ca1-xSrxZr4P6O24, x = 0.0							
File Name	Temp -erature (℃)	Temp -erature ESD (℃)	*a*-Axis Unit-cell Parameter (Å)	*a*-Axis Unit-cell Paramete r ESD (Å)	*c*-Axis Unit-cell Parameter (Å)	*c*-Axis Unit-cell Paramete r ESD (Å)	Space Group
JB1007A_01_25	25	4	8.7838	0.0002	22.7086	0.0010	R-3 (148)
JB1007A_02_25	25	4	8.7839	0.0002	22.7069	0.0010	R-3 (148)
JB1007A_03_25	23	3	8.7841	0.0002	22.7067	0.0010	R-3 (148)
JB1007A_04_200	349	3	8.7700	0.0002	22.7829	0.0010	R-3 (148)
JB1007A_05_300	469	3	8.7683	0.0002	22.8114	0.0010	R-3 (148)
JB1007A_06_400	607	3	8.7671	0.0002	22.8456	0.0010	R-3 (148)
JB1007A_07_540 0	728	3	8.7657	0.0002	22.8754	0.0010	R-3 (148)
JB1007A_08_600	828	3	8.7643	0.0002	22.9013	0.0010	R-3 (148)
JB1007A_09_700	908	2	8.7631	0.0002	22.9229	0.0010	R-3 (148)
JB1007A_10_800	951	2	8.7623	0.0002	22.9354	0.0010	R-3 (148)
JB1007A_10_800	990	2	8.7620	0.0002	22.9448	0.0009	R-3 (148)
JB1007A_11_900	1046	2	8.7621	0.0002	22.9573	0.0009	R-3 (148)
JB1007A_12_100 0	1099	2	8.7622	0.0002	22.9707	0.0009	R-3 (148)
JB1007A_13_110 0	1163	2	8.7626	0.0002	22.9874	0.0009	R-3 (148)
JB1007A_14_120 0	1268	2	8.7617	0.0002	23.0153	0.0009	R-3c (167)
JB1007A_15_130 0	1330	2	8.7606	0.0002	23.0326	0.0009	R-3c (167)
JB1007A_16_140 0	1420	2	8.7588	0.0002	23.0569	0.0010	R-3c (167)
JB1007A_17_150 0	1361	2	8.7599	0.0002	23.0427	0.0009	R-3c (167)
JB1007A_18_140 0	1297	2	8.7611	0.0002	23.0268	0.0009	R-3c (167)
JB1007A_19_130 0	1231	2	8.7623	0.0002	23.0100	0.0009	R-3c (167)
JB1007A_20_120 0	1163	2	8.7630	0.0002	22.9930	0.0009	R-3c (167)
JB1007A_21_110 0	1094	2	8.7620	0.0002	22.9773	0.0009	R-3 (148)
JB1007A_22_100 0	1022	2	8.7616	0.0002	22.9611	0.0009	R-3 (148)
JB1007A_23_900	942	3	8.7614	0.0002	22.9428	0.0009	R-3 (148)
JB1007A_24_800	857	3	8.7617	0.0002	22.9245	0.0010	R-3 (148)

JB1007A_25_700	763	3	8.7622	0.0002	22.9053	0.0010	R-3 (148)
JB1007A_26_600	654	3	8.7632	0.0002	22.8829	0.0010	R-3 (148)
JB1007A_27_500	549	3	8.7643	0.0002	22.8622	0.0010	R-3 (148)
JB1007A_28_400	421	3	8.7665	0.0002	22.8353	0.0011	R-3 (148)
JB1007A_29_300	295	4	8.7707	0.0003	22.8035	0.0011	R-3 (148)
JB1007A_30_200	37	4	8.7877	0.0003	22.7199	0.0011	R-3 (148)

Table S6 Ca1-xSrxZr4P6O24, x = 1.0 unit-cell parameter data

JB1008A_APS_17-BM-B_2020: Ca1-xSrxZr4P6O24, x = 1.0							
File Name	Temp -erature (℃)	Temp -erature ESD (℃)	*a*-Axis Unit-cell Parameter (Å)	*a*-Axis Unit-cell Paramete r ESD (Å)	*c*-Axis Unit-cell Parameter (Å)	*c*-Axis Unit-cell Paramete r ESD (Å)	Space Group
JB1008A_01_25	25	4	8.6969	0.0002	23.3671	0.0010	R-3 (148)
JB1008A_02_25	22	4	8.6968	0.0002	23.3670	0.0010	R-3 (148)
JB1008A_03_300	576	3	8.7089	0.0002	23.3908	0.0010	R-3 (148)
JB1008A_04_400	710	3	8.7112	0.0002	23.3995	0.0010	R-3 (148)
JB1008A_05_500	8218	3	8.7131	0.0002	23.4067	0.0010	R-3 (148)
JB1008A_06_600	916	3	8.7150	0.0002	23.4134	0.0010	R-3 (148)
JB1008A_07_700	979	3	8.7163	0.0002	23.4177	0.0010	R-3 (148)
JB1008A_08_800	1020	3	8.7172	0.0002	23.4205	0.0011	R-3 (148)
JB1008A_09_900	1071	3	8.7185	0.0002	23.4236	0.0011	R-3 (148)
JB1008A_10_1000	1128	3	8.7202	0.0002	23.4269	0.0011	R-3 (148)
JB1008A_11_1100	1182	3	8.7224	0.0002	23.4287	0.0011	R-3 (148)
JB1008A_12_1200	1262	4	8.7272	0.0004	23.4291	0.0014	R-3c (167)
JB1008A_13_1300	1335	4	8.7275	0.0004	23.4372	0.0015	R-3c (167)
JB1008A_14_1400	1409	4	8.7269	0.0004	23.4488	0.0015	R-3c (167)
JB1008A_15_1500	1490	4	8.7258	0.0004	23.4623	0.0015	R-3c (167)
JB1008A_16_1400	1429	4	8.7268	0.0004	23.4519	0.0015	R-3c (167)
JB1008A_17_1300	1363	5	8.7276	0.0004	23.4410	0.0015	R-3c (167)
JB1008A_18_1200	1295	5	8.7283	0.0004	23.4307	0.0015	R-3c (167)

JB1008A_19_1100	1227	5	8.7278	0.0004	23.4239	0.0014	R-3c (167)
JB1008A_20_1000	1145	4	8.7233	0.0002	23.4223	0.0011	R-3 (148)
JB1008A_21_900	1065	4	8.7204	0.0002	23.4184	0.0011	R-3 (148)
JB1008A_22_800	984	4	8.7183	0.0002	23.4130	0.0011	R-3 (148)
JB1008A_23_700	890	4	8.7163	0.0002	23.4063	0.0011	R-3 (148)
JB1008A_24_600	794	4	8.7145	0.0002	23.3997	0.0011	R-3 (148)
JB1008A_25_500	678	4	8.7125	0.0002	23.3916	0.0011	R-3 (148)
JB1008A_26_400	561	4	8.7105	0.0002	23.3839	0.0011	R-3 (148)
JB1008A_27_300	433	4	8.7081	0.0002	23.3758	0.0011	R-3 (148)
JB1008A_28_25	24	4	8.6997	0.0002	23.3571	0.0010	R-3 (148)

Table S7 Ca1-xSrxZr4P6O24, x = 0.5 unit-cell parameter data

JB1011A_APS_17-BM-B_2020: Ca1-xSrxZr4P6O24, x = 0.5							
File Name	Temp-erature (°C)	Temp-erature ESD (°C)	*a*-Axis Unit-cell Parameter (Å)	*a*-Axis Unit-cell Parameter ESD (Å)	*c*-Axis Unit-cell Parameter (Å)	*c*-Axis Unit-cell Parameter ESD (Å)	Space Group
JB1011A_01_25	25	7	8.7253	0.0005	23.1019	0.0005	R-3 (148)
JB1011A_02_25	23	8	8.7250	0.0005	23.1035	0.0005	R-3 (148)
JB1011A_03_200	359	7	8.7271	0.0004	23.1298	0.0004	R-3 (148)
JB1011A_04_300	504	6	8.7287	0.0004	23.1449	0.0004	R-3 (148)
JB1011A_05_400	641	6	8.7299	0.0004	23.1587	0.0004	R-3 (148)
JB1011A_06_500	746	6	8.7308	0.0004	23.1705	0.0004	R-3 (148)
JB1011A_07_600	849	5	8.7318	0.0004	23.1822	0.0004	R-3 (148)
JB1011A_08_700	933	5	8.7328	0.0004	23.1929	0.0004	R-3 (148)
JB1011A_09_800	1001	5	8.7337	0.0004	23.2018	0.0004	R-3 (148)
JB1011A_10_900	1064	5	8.7347	0.0004	23.2102	0.0004	R-3 (148)
JB1011A_11_1000	1122	5	8.7358	0.0004	23.2173	0.0004	R-3 (148)
JB1011A_12_1100	1173	5	8.7370	0.0005	23.2233	0.0005	R-3 (148)
JB1011A_13_1200	1223	5	8.7387	0.0005	23.2288	0.0005	R-3 (148)
JB1011A_14_130	1296	5	8.7421	0.0005	23.2339	0.0005	R-3

0							(148)
JB1011A_15_1400	1344	6	8.7430	0.0006	23.2396	0.0006	R-3c (167)
JB1011A_16_1500	1411	6	8.7418	0.0006	23.2510	0.0006	R-3c (167)
JB1011A_17_1400	1360	6	8.7427	0.0006	23.2431	0.0006	R-3c (167)
JB1011A_18_1300	1307	6	8.7434	0.0006	23.2347	0.0006	R-3c (167)
JB1011A_19_1200	1249	5	8.7408	0.0005	23.2308	0.0005	R-3 (148)
JB1011A_20_1100	1183	5	8.7383	0.0004	23.2246	0.0004	R-3 (148)
JB1011A_21_1000	1110	5	8.7362	0.0004	23.2170	0.0004	R-3 (148)
JB1011A_22_900	1026	5	8.7345	0.0004	23.2058	0.0004	R-3 (148)
JB1011A_23_800	932	5	8.7332	0.0004	23.1935	0.0004	R-3 (148)
JB1011A_24_700	831	5	8.7320	0.0004	23.1812	0.0004	R-3 (148)
JB1011A_25_600	730	6	8.7310	0.0004	23.1697	0.0004	R-3 (148)
JB1011A_26_500	622	6	8.7300	0.0004	23.1584	0.0004	R-3 (148)
JB1011A_27_400	504	6	8.7290	0.0004	23.1470	0.0004	R-3 (148)
JB1011A_28_300	385	6	8.7279	0.0004	23.1364	0.0004	R-3 (148)
JB1011A_29_200	267	6	8.7269	0.0004	23.1261	0.0004	R-3 (148)

Table S8 Ca1-xSrxZr4P6O24, x = 0.4 unit-cell parameter data

JB1013A_APS_17-BM-B_2020: Ca1-xSrxZr4P6O24, x = 0.4							
File Name	Temp -erature (℃)	Temp -erature ESD (℃)	*a*-Axis Unit-cell Parameter (Å)	*a*-Axis Unit-cell Paramete r ESD (Å)	*c*-Axis Unit-cell Parameter (Å)	*c*-Axis Unit-cell Paramete r ESD (Å)	Space Group
JB1013A_01_25	25	7	8.7319	0.0005	23.0522	0.0005	R-3 (148)
JB1013A_02_25	24	7	8.7319	0.0005	23.0522	0.0005	R-3 (148)
JB1013A_03_200	415	7	8.7366	0.0005	23.0952	0.0005	R-3 (148)
JB1013A_04_300	573	7	8.7396	0.0006	23.1196	0.0006	R-3 (148)
JB1013A_05_400	749	6	8.7424	0.0006	23.1463	0.0006	R-3 (148)
JB1013A_06_500	884	6	8.7447	0.0006	23.1656	0.0006	R-3 (148)
JB1013A_07_600	990	6	8.7464	0.0006	23.1812	0.0006	R-3 (148)
JB1013A_08_700	1031	6	8.7477	0.0005	23.1871	0.0005	R-3 (148)
JB1013A_09_800	1070	5	8.7485	0.0005	23.1920	0.0005	R-3

							(148)
JB1013A_10_900	1131	6	8.7494	0.0006	23.2009	0.0006	R-3 (148)
JB1013A_11_1000	1190	6	8.7501	0.0006	23.2096	0.0006	R-3 (148)
JB1013A_12_1100	1254	6	8.7495	0.0006	23.2195	0.0006	R-3c (167)
JB1013A_13_1200	1315	6	8.7485	0.0006	23.2283	0.0006	R-3c (167)
JB1013A_14_1300	1392	6	8.7469	0.0006	23.2453	0.0006	R-3c (167)
JB1013A_15_1400	1473	6	8.7456	0.0006	23.2646	0.0006	R-3c (167)
JB1013A_16_1500	1543	6	8.7450	0.0006	23.2844	0.0006	R-3c (167)
JB1013A_17_1400	1480	6	8.7460	0.0006	23.2754	0.0006	R-3c (167)
JB1013A_18_1300	1422	6	8.7473	0.0006	23.2651	0.0006	R-3c (167)
JB1013A_19_1200	1355	6	8.7485	0.0006	23.2525	0.0006	R-3c (167)
JB1013A_20_1100	1283	5	8.7498	0.0005	23.2386	0.0005	R-3c (167)
JB1013A_21_1000	1205	6	8.7513	0.0006	23.2229	0.0006	R-3c (167)
JB1013A_22_900	1119	6	8.7525	0.0006	23.2088	0.0006	R-3c (167)
JB1013A_23_800	1023	6	8.7536	0.0006	23.1888	0.0006	R-3c (167)
JB1013A_24_700	920	6	8.7536	0.0005	23.1708	0.0005	R-3 (148)
JB1013A_25_600	810	6	8.7520	0.0005	23.1543	0.0005	R-3 (148)
JB1013A_26_500	679	6	8.7502	0.0005	23.1346	0.0005	R-3 (148)
JB1013A_27_400	547	6	8.7491	0.0005	23.1158	0.0005	R-3 (148)
JB1013A_28_300	425	6	8.7492	0.0005	23.0978	0.0005	R-3 (148)
JB1013A_29_200	296	7	8.7501	0.0005	23.0749	0.0005	R-3 (148)
JB1013A_30_25	33	8	8.7570	0.0006	23.0219	0.0006	R-3 (148)

Table S9 Ca1-xSrxZr4P6O24, x = 0.2 unit-cell parameter data

JB1012A_APS_17-BM-B_2020: Ca1-xSrxZr4P6O24, x = 0.2							
File Name	Temp -erature (°C)	Temp -erature ESD (°C)	*a*-Axis Unit-cell Parameter (Å)	*a*-Axis Unit-cell Parameter ESD (Å)	*c*-Axis Unit-cell Parameter (Å)	*c*-Axis Unit-cell Parameter ESD (Å)	Space Group
JB1012A_01_25	25	10	8.7486	0.0006	22.9045	0.0006	R-3 (148)
JB1012A_02_25	25	10	8.7486	0.0006	22.9045	0.0006	R-3 (148)
JB1012A_03_200	355	7	8.7465	0.0005	22.9414	0.0005	R-3

							(148)
JB1012A_04_300	505	8	8.7467	0.0005	22.9615	0.0005	R-3 (148)
JB1012A_05_400	626	8	8.7461	0.0005	22.9778	0.0005	R-3 (148)
JB1012A_06_500	732	8	8.7460	0.0005	22.9928	0.0005	R-3 (148)
JB1012A_07_600	831	8	8.7460	0.0005	23.0063	0.0005	R-3 (148)
JB1012A_08_700	913	8	8.7459	0.0005	23.0170	0.0005	R-3 (148)
JB1012A_09_800	979	8	8.7462	0.0005	23.0269	0.0005	R-3 (148)
JB1012A_10_900	1040	8	8.7466	0.0005	23.0365	0.0005	R-3 (148)
JB1012A_11_1000	1102	8	8.7472	0.0005	23.0473	0.0005	R-3 (148)
JB1012A_12_1100	1160	8	8.7477	0.0005	23.0577	0.0005	R-3 (148)
JB1012A_13_1200	1217	8	8.7488	0.0005	23.0683	0.0005	R-3 (148)
JB1012A_14_1300	1277	9	8.7504	0.0005	23.0786	0.0005	R-3 (148)
JB1012A_15_1400	1344	9	8.7533	0.0006	23.0896	0.0006	R-3c (167)
JB1012A_16_1500	1414	8	8.7518	0.0006	23.1075	0.0006	R-3c (167)
JB1012A_17_1400	1362	9	8.7528	0.0006	23.0973	0.0006	R-3c (167)
JB1012A_18_1300	1307	9	8.7526	0.0005	23.0870	0.0005	R-3 (148)
JB1012A_19_1200	1248	9	8.7506	0.0005	23.0785	0.0005	R-3 (148)
JB1012A_20_1100	1180	8	8.7489	0.0005	23.0668	0.0005	R-3 (148)
JB1012A_21_1000	1107	8	8.7477	0.0005	23.0548	0.0005	R-3 (148)
JB1012A_22_900	1023	8	8.7466	0.0005	23.0409	0.0005	R-3 (148)
JB1012A_23_800	928	8	8.7458	0.0005	23.0270	0.0005	R-3 (148)
JB1012A_24_700	830	8	8.7455	0.0005	23.0148	0.0005	R-3 (148)
JB1012A_25_600	732	8	8.7453	0.0005	23.0027	0.0005	R-3 (148)
JB1012A_26_500	623	8	8.7452	0.0005	22.9884	0.0005	R-3 (148)
JB1012A_27_400	504	7	8.7450	0.0005	22.9734	0.0005	R-3 (148)
JB1012A_28_300	386	7	8.7450	0.0005	22.9584	0.0005	R-3 (148)
JB1012A_29_200	266	7	8.7446	0.0005	22.9428	0.0005	R-3 (148)
JB1012A_30_25	21	7	8.7464	0.0006	22.9161	0.0006	R-3 (148)

Table S10 Ca1-xSrxZr4P6O24, x = 0.8 unit-cell parameter data

JB1009A_APS_17-BM-B_2020: Ca1-xSrxZr4P6O24, x = 0.8							
File Name	Temp -erature (℃)	Temp -erature ESD (℃)	*a*-Axis Unit-cell Parameter (Å)	*a*-Axis Unit-cell Paramete r ESD (Å)	*c*-Axis Unit-cell Parameter (Å)	*c*-Axis Unit-cell Paramete r ESD (Å)	Space Group
JB1009A_01_25	25	6	8.7050	0.0005	23.2943	0.0005	R-3 (148)
JB1009A_02_25	27	6	8.7051	0.0005	23.2946	0.0005	R-3 (148)
JB1009A_03_25	26	6	8.7051	0.0005	23.2946	0.0005	R-3 (148)
JB1009A_04_200	387	5	8.7120	0.0005	23.3130	0.0005	R-3 (148)
JB1009A_05_300	529	5	8.7143	0.0005	23.3227	0.0005	R-3 (148)
JB1009A_06_400	656	4	8.7163	0.0005	23.3319	0.0005	R-3 (148)
JB1009A_07_500	777	4	8.7183	0.0005	23.3422	0.0005	R-3 (148)
JB1009A_08_600	878	4	8.7199	0.0005	23.3507	0.0005	R-3 (148)
JB1009A_09_700	968	4	8.7215	0.0005	23.3583	0.0005	R-3 (148)
JB1009A_10_800	1042	4	8.7233	0.0005	23.3643	0.0005	R-3 (148)
JB1009A_11_900	1108	4	8.7250	0.0005	23.3689	0.0005	R-3 (148)
JB1009A_12_1000	1174	4	8.7275	0.0005	23.3727	0.0005	R-3 (148)
JB1009A_13_1100	1268	5	8.7319	0.0007	23.3750	0.0007	R-3 (148)
JB1009A_14_1200	1293	5	8.7324	0.0007	23.3785	0.0007	R-3 (148)
JB1009A_15_1300	1363	5	8.7314	0.0007	23.3897	0.0007	R-3c (167)
JB1009A_16_1400	1441	5	8.7303	0.0007	23.4038	0.0007	R-3c (167)
JB1009A_17_1500	1535	5	8.7298	0.0007	23.4215	0.0007	R-3c (167)
JB1009A_18_1400	1479	5	8.7310	0.0007	23.4150	0.0007	R-3c (167)
JB1009A_19_1300	1419	5	8.7321	0.0007	23.4054	0.0007	R-3c (167)
JB1009A_20_1200	1351	5	8.7325	0.0007	23.3933	0.0007	R-3c (167)
JB1009A_21_1100	1276	5	8.7335	0.0007	23.3808	0.0007	R-3c (167)
JB1009A_22_1000	1194	5	8.7331	0.0006	23.3698	0.0006	R-3c (167)
JB1009A_23_900	1102	4	8.7291	0.0005	23.3658	0.0005	R-3 (148)
JB1009A_24_800	1002	4	8.7261	0.0005	23.3588	0.0005	R-3 (148)
JB1009A_25_700	897	4	8.7238	0.0005	23.3499	0.0005	R-3 (148)

JB1009A_26_600	787	4	8.7219	0.0005	23.3404	0.0005	R-3 (148)
JB1009A_27_500	665	4	8.7200	0.0005	23.3299	0.0005	R-3 (148)
JB1009A_28_400	544	5	8.7181	0.0005	23.3203	0.0005	R-3 (148)
JB1009A_29_300	415	5	8.7160	0.0005	23.3107	0.0005	R-3 (148)
JB1009A_30_200	293	5	8.7138	0.0005	23.3022	0.0005	R-3 (148)
JB1009A_31_25	44	6	8.7105	0.0005	23.2899	0.0005	R-3 (148)

Table S11 Ca1-xSrxZr4P6O24, x = 0.6 unit-cell parameter data

JB1010A_APS_17-BM-B_2020: Ca1-xSrxZr4P6O24, x = 0.6							
File Name	Temp -erature (°C)	Temp -erature ESD (°C)	*a*-Axis Unit-cell Parameter (Å)	*a*-Axis Unit-cell Paramete r ESD (Å)	*c*-Axis Unit-cell Parameter (Å)	*c*-Axis Unit-cell Paramete r ESD (Å)	Space Group
JB1010A_01_25	25	6	8.7200	0.0005	23.1559	0.0005	R-3 (148)
JB1010A_02_25	26	6	8.7200	0.0005	23.1561	0.0005	R-3 (148)
JB1010A_03_25	26	6	8.7200	0.0005	23.1561	0.0005	R-3 (148)
JB1010A_04_200	344	6	8.7229	0.0004	23.1778	0.0004	R-3 (148)
JB1010A_05_300	475	6	8.7246	0.0004	23.1909	0.0004	R-3 (148)
JB1010A_06_400	597	5	8.7257	0.0004	23.2020	0.0004	R-3 (148)
JB1010A_07_500	701	5	8.7268	0.0004	23.2124	0.0004	R-3 (148)
JB1010A_08_600	804	4	8.7279	0.0004	23.2226	0.0004	R-3 (148)
JB1010A_09_700	892	4	8.7291	0.0004	23.2318	0.0004	R-3 (148)
JB1010A_10_800	963	4	8.7303	0.0004	23.2400	0.0004	R-3 (148)
JB1010A_11_900	1033	4	8.7315	0.0004	23.2479	0.0004	R-3 (148)
JB1010A_12_1000	1102	4	8.7332	0.0004	23.2557	0.0004	R-3 (148)
JB1010A_13_1100	1164	4	8.7350	0.0004	23.2624	0.0004	R-3 (148)
JB1010A_14_1200	1226	4	8.7376	0.0005	23.2685	0.0005	R-3 (148)
JB1010A_15_1300	1290	5	8.7405	0.0006	23.2739	0.0006	R-3 (148)
JB1010A_16_1400	1364	5	8.7398	0.0006	23.2865	0.0006	R-3c (167)
JB1010A_17_1500	1447	5	8.7383	0.0006	23.3011	0.0006	R-3c (167)
JB1010A_18_1400	1388	5	8.7395	0.0006	23.2915	0.0006	R-3c (167)

JB1010A_19_1300	1328	5	8.7406	0.0006	23.2817	0.0006	R-3c (167)
JB1010A_20_1200	1263	5	8.7411	0.0006	23.2711	0.0006	R-3c (167)
JB1010A_21_1100	1188	4	8.7376	0.0004	23.2650	0.0004	R-3 (148)
JB1010A_22_1000	1105	4	8.7346	0.0004	23.2579	0.0004	R-3 (148)
JB1010A_23_900	1010	4	8.7322	0.0004	23.2471	0.0004	R-3 (148)
JB1010A_24_800	909	4	8.7301	0.0004	23.2358	0.0004	R-3 (148)
JB1010A_25_700	806	4	8.7286	0.0004	23.2255	0.0004	R-3 (148)
JB1010A_26_600	702	5	8.7274	0.0004	23.2155	0.0004	R-3 (148)
JB1010A_27_500	587	5	8.7262	0.0004	23.2051	0.0004	R-3 (148)
JB1010A_28_400	480	5	8.7249	0.0004	23.1955	0.0004	R-3 (148)
JB1010A_29_300	366	5	8.7235	0.0004	23.1859	0.0004	R-3 (148)
JB1010A_30_200	258	5	8.7222	0.0004	23.1774	0.0004	R-3 (148)
JB1010A_31_25	43	6	8.7208	0.0004	23.1653	0.0004	R-3 (148)

Table S12 Ca1-xSrxZr4P6O24, x = 0.0 unit-cell parameter data

JB1007A_quartz2_APS_17-BM-B: Ca1-xSrxZr4P6O24, x = 0.0							
File Name	Temp - erature (°C)	Temp - erature ESD (°C)	*a*-Axis Unit-cell Paramet er (Å)	*a*-Axis Unit-cell Paramet er ESD (Å)	*c*-Axis Unit-cell Paramet er (Å)	*c*-Axis Unit- cell Paramet er ESD (Å)	Space Group
JB1010A_01_25	25	5	8.7850	0.0003	22.7034	0.0003	R-3 (148)
JB1010A_02_25	21	5	8.7850	0.0003	22.7028	0.0003	R-3 (148)
JB1010A_03_25	208	4	8.7763	0.0003	22.7415	0.0003	R-3 (148)
JB1010A_04_200	258	4	8.7742	0.0003	22.7527	0.0003	R-3 (148)
JB1010A_05_300	341	4	8.7709	0.0003	22.7721	0.0003	R-3 (148)
JB1010A_06_400	412	3	8.7678	0.0003	22.7911	0.0003	R-3 (148)
JB1010A_07_500	489	3	8.7655	0.0003	22.8077	0.0003	R-3 (148)
JB1010A_08_600	558	3	8.7638	0.0003	22.8220	0.0003	R-3 (148)
JB1010A_09_700	593	3	8.7631	0.0003	22.8296	0.0003	R-3 (148)
JB1010A_10_800	629	3	8.7624	0.0003	22.8374	0.0003	R-3 (148)
JB1010A_11_900	664	3	8.7617	0.0003	22.8449	0.0003	R-3 (148)

JB1010A_12_1000	698	2	8.7612	0.0003	22.8524	0.0003	R-3 (148)
JB1010A_13_1100	733	2	8.7606	0.0003	22.8601	0.0003	R-3 (148)
JB1010A_14_1200	768	2	8.7601	0.0003	22.8675	0.0003	R-3 (148)
JB1010A_15_1300	800	2	8.7595	0.0003	22.8745	0.0003	R-3 (148)
JB1010A_16_1400	835	2	8.7589	0.0003	22.8818	0.0003	R-3 (148)
JB1010A_17_1500	869	2	8.7584	0.0003	22.8891	0.0003	R-3 (148)

Table S13 $NaTi_2(PO_4)_3$ Temperature Dependent Unit-Cell Data

BSH2085A_2 NSLS II 28-ID-2: NaTi2(PO4)3							
File Name	Temperature (°C)	Temperature ESD (°C)	*a*-Axis Unit-cell Parameter (Å)	*a*-Axis Unit-cell Parameter ESD (Å)	*c*-Axis Unit-cell Parameter (Å)	*c*-Axis Unit-cell Parameter ESD (Å)	Space Group
BSH2085A_2_20211031-022744	25	1	8.4931	0.0001	21.8016	0.0006	R-3c (167)
BSH2085A_2_20211031-023441	23	1	8.4933	0.0001	21.8004	0.0006	R-3c (167)
BSH2085A_2_20211031-024001	99	1	8.4892	0.0001	21.8355	0.0006	R-3c (167)
BSH2085A_2_20211031-024241	257	1	8.4811	0.0001	21.9049	0.0006	R-3c (167)
BSH2085A_2_20211031-024519	402	1	8.4756	0.0001	21.9701	0.0006	R-3c (167)
BSH2085A_2_20211031-024756	526	2	8.4709	0.0002	22.0256	0.0007	R-3c (167)
BSH2085A_2_20211031-025032	634	2	8.4681	0.0001	22.0735	0.0007	R-3c (167)
BSH2085A_2_20211031-025309	744	2	8.4655	0.0002	22.1209	0.0008	R-3c (167)
BSH2085A_2_20211031-025615	799	2	8.4640	0.0002	22.1425	0.0007	R-3c (167)
BSH2085A_2_20211031-025843	849	2	8.4628	0.0002	22.1638	0.0008	R-3c (167)
BSH2085A_2_20211031-030111	907	2	8.4617	0.0002	22.1884	0.0008	R-3c (167)
BSH2085A_2_20211031-030339	954	2	8.4616	0.0002	22.2068	0.0008	R-3c (167)
BSH2085A_2_20211031-030607	1008	2	8.4613	0.0002	22.2250	0.0008	R-3c (167)
BSH2085A_2_20211031-030836	1055	2	8.4608	0.0002	22.2458	0.0008	R-3c (167)
BSH2085A_2_20211031-031105	1095	2	8.4616	0.0002	22.2622	0.0008	R-3c (167)
BSH2085A_2_20211031-031335	1155	2	8.4614	0.0002	22.2815	0.0008	R-3c (167)
BSH2085A_2_20211031-031604	1209	2	8.4618	0.0002	22.2991	0.0010	R-3c (167)
BSH2085A_2_20211031-031833	1263	2	8.4630	0.0002	22.3174	0.0008	R-3c (167)
BSH2085A_2_20211031-032103	1307	2	8.4642	0.0002	22.3353	0.0010	R-3c (167)
BSH2085A_2_20211031-032333	1368	2	8.4661	0.0002	22.3535	0.0009	R-3c (167)
BSH2085A_2_20211031-032601	1425	2	8.4696	0.0002	22.3760	0.0009	R-3c (167)

Table S14 $NaZr_2(PO_4)_3$ Temperature Dependent Unit-Cell Data

BSH2084A NSLS II 28-ID-2: NaZr2(PO4)3							
File Name	Temp - erature (°C)	Temp - erature ESD (°C)	*a*-Axis Unit-cell Parameter (Å)	*a*-Axis Unit-cell Parameter ESD (Å)	*c*-Axis Unit-cell Parameter (Å)	*c*-Axis Unit-cell Parameter ESD (Å)	Space Group
BSH2084A_20211029-193525	25	6	8.8014	0.0001	22.7551	0.0001	R-3c (167)
BSH2084A_20211029-193841	25	6	8.8014	0.0001	22.7552	0.0001	R-3c (167)
BSH2084A_20211029-201703	85	6	8.7974	0.0001	22.7899	0.0001	R-3c (167)
BSH2084A_20211029-201941	239	7	8.7885	0.0001	22.8763	0.0001	R-3c (167)
BSH2084A_20211029-202223	396	7	8.7806	0.0002	22.9607	0.0002	R-3c (167)
BSH2084A_20211029-202458	516	8	8.7744	0.0002	23.0227	0.0002	R-3c (167)
BSH2084A_20211029-202733	629	8	8.7701	0.0002	23.0717	0.0002	R-3c (167)
BSH2084A_20211029-203008	740	8	8.7660	0.0002	23.1136	0.0002	R-3c (167)
BSH2084A_20211029-203402	777	8	8.7643	0.0002	23.1328	0.0002	R-3c (167)
BSH2084A_20211029-203627	836	8	8.7628	0.0002	23.1502	0.0002	R-3c (167)
BSH2084A_20211029-203852	880	8	8.7613	0.0002	23.1683	0.0002	R-3c (167)
BSH2084A_20211029-204118	932	9	8.7602	0.0002	23.1839	0.0002	R-3c (167)
BSH2084A_20211029-204344	971	8	8.7591	0.0002	23.1992	0.0002	R-3c (167)
BSH2084A_20211029-204609	1015	9	8.7579	0.0002	23.2151	0.0002	R-3c (167)
BSH2084A_20211029-204835	1059	8	8.7573	0.0002	23.2286	0.0002	R-3c (167)
BSH2084A_20211029-205103	1111	9	8.7567	0.0002	23.2402	0.0002	R-3c (167)
BSH2084A_20211029-205331	1158	8	8.7559	0.0002	23.2535	0.0002	R-3c (167)
BSH2084A_20211029-205601	1194	9	8.7554	0.0002	23.2652	0.0002	R-3c (167)
BSH2084A_20211029-205831	1239	10	8.7548	0.0003	23.2748	0.0003	R-3c (167)
BSH2084A_20211029-210101	1303	10	8.7543	0.0003	23.2857	0.0003	R-3c (167)
BSH2084A_20211029-210330	1340	10	8.7526	0.0003	23.2930	0.0003	R-3c (167)
BSH2084A_20211029-210601	1378	10	8.7518	0.0003	23.3046	0.0003	R-3c (167)
BSH2084A_20211029-210848	1343	13	8.7519	0.0003	23.3056	0.0003	R-3c (167)
BSH2084A_20211029-211129	1284	13	8.7523	0.0003	23.2819	0.0003	R-3c (167)
BSH2084A_20211029-	1206	10	8.7533	0.0003	23.2596	0.0003	R-3c

211409							(167)
BSH2084A_20211029-211647	1079	8	8.7542	0.0003	23.2393	0.0003	R-3c (167)
BSH2084A_20211029-211922	985	8	8.7555	0.0003	23.2147	0.0003	R-3c (167)
BSH2084A_20211029-212157	892	7	8.7575	0.0003	23.1866	0.0003	R-3c (167)
BSH2084A_20211029-212431	831	6	8.7596	0.0002	23.1563	0.0002	R-3c (167)
BSH2084A_20211029-212705	734	5	8.7626	0.0002	23.1218	0.0002	R-3c (167)
BSH2084A_20211029-212940	567	6	8.7656	0.0002	23.0820	0.0002	R-3c (167)
BSH2084A_20211029-213215	421	5	8.7702	0.0002	23.0343	0.0002	R-3c (167)
BSH2084A_20211029-213452	357	4	8.7761	0.0002	22.9720	0.0002	R-3c (167)
BSH2084A_20211029-213731	185	4	8.7851	0.0002	22.8758	0.0002	R-3c (167)
BSH2084A_20211029-214011	30	4	8.7931	0.0002	22.7977	0.0002	R-3c (167)
BSH2084A_20211029-214816	0	4	8.7960	0.0002	22.7714	0.0002	R-3c (167)

Table S15 $MgZr_4(PO_4)_6$ Temperature Dependent Unit-Cell Data

JB1015A APS 17-BM-B: Mg0.5Zr2(PO4)3											
File Name	Temp-erature (°C)	Temp-erature ESD (°C)	*a*-Axis Unit-cell Parameter (Å)	*a*-Axis Unit-cell Parameter ESD (Å)	*b*-Axis Unit-cell Parameter (Å)	*b*-Axis Unit-cell Parameter ESD (Å)	*c*-Axis Unit-cell Parameter (Å)	*c*-Axis Unit-cell Parameter ESD (Å)	β Angle (Å)	β Angle ESD (Å)	Space Group
JB1015A_01_25	25	9	12.4295	0.0007	8.9190	0.0007	8.8266	0.0007	90.5188	0.0007	$P2_1/n$ (14)
JB1015A_02_25	22	8	12.4294	0.0007	8.9187	0.0007	8.8265	0.0007	90.5184	0.0007	$P2_1/n$ (14)
JB1015A_03_200	262	8	12.4281	0.0007	8.9351	0.0007	8.8285	0.0007	90.5233	0.0007	$P2_1/n$ (14)
JB1015A_04_300	336	7	12.4277	0.0007	8.9398	0.0007	8.8300	0.0007	90.5163	0.0007	$P2_1/n$ (14)
JB1015A_05_400	396	7	12.4274	0.0007	8.9429	0.0007	8.8309	0.0007	90.5073	0.0007	$P2_1/n$ (14)
JB1015A_06_500	461	7	12.4271	0.0007	8.9458	0.0007	8.8321	0.0007	90.4920	0.0007	$P2_1/n$ (14)
JB1015A_07_600	527	6	12.4266	0.0008	8.9482	0.0008	8.8333	0.0008	90.4740	0.0008	$P2_1/n$ (14)
JB1015A_08_650	557	6	12.4266	0.0008	8.9490	0.0008	8.8340	0.0008	90.4644	0.0008	$P2_1/n$ (14)
JB1015A_09_700	586	6	12.4267	0.0008	8.9497	0.0008	8.8347	0.0008	90.4534	0.0008	$P2_1/n$ (14)
JB1015A_10_750	615	6	12.4268	0.0008	8.9500	0.0008	8.8354	0.0008	90.4411	0.0008	$P2_1/n$ (14)
JB1015A_11_800	646	6	12.4273	0.0008	8.9502	0.0008	8.8363	0.0008	90.4253	0.0008	$P2_1/n$ (14)

JB1015A_12_850	680	6	12.4283	0.0008	8.9502	0.0008	8.8376	0.0008	90.4042	0.0008	$P2_1/n$ (14)
JB1015A_13_900	720	5	12.4304	0.0009	8.9499	0.0009	8.8395	0.0009	90.3750	0.0009	$P2_1/n$ (14)
JB1015A_14_950	766	5	12.4338	0.0009	8.9493	0.0009	8.8424	0.0009	90.3304	0.0009	$P2_1/n$ (14)
JB1015A_15_1000	812	5	12.4380	0.0008	8.9483	0.0008	8.8460	0.0008	90.2672	0.0008	$P2_1/n$ (14)
JB1015A_16_1050	865	5	12.4440	0.0008	8.9465	0.0008	8.8513	0.0008	90.1298	0.0008	$P2_1/n$ (14)
JB1015A_17_1000	845	5	12.4418	0.0008	8.9471	0.0008	8.8495	0.0008	90.1885	0.0008	$P2_1/n$ (14)
JB1015A_18_950	813	5	12.4385	0.0009	8.9481	0.0009	8.8466	0.0009	90.2577	0.0009	$P2_1/n$ (14)
JB1015A_19_900	776	5	12.4352	0.0009	8.9489	0.0009	8.8436	0.0009	90.3118	0.0009	$P2_1/n$ (14)
JB1015A_20_850	739	6	12.4324	0.0009	8.9496	0.0009	8.8412	0.0009	90.3516	0.0009	$P2_1/n$ (14)
JB1015A_21_800	700	6	12.4298	0.0009	8.9498	0.0009	8.8390	0.0009	90.3832	0.0009	$P2_1/n$ (14)
JB1015A_22_750	663	6	12.4280	0.0009	8.9500	0.0009	8.8374	0.0009	90.4099	0.0009	$P2_1/n$ (14)
JB1015A_23_700	625	6	12.4271	0.0009	8.9500	0.0009	8.8361	0.0009	90.4324	0.0009	$P2_1/n$ (14)
JB1015A_24_650	587	6	12.4268	0.0008	8.9497	0.0008	8.8350	0.0008	90.4510	0.0008	$P2_1/n$ (14)
JB1015A_25_600	548	6	12.4267	0.0008	8.9488	0.0008	8.8339	0.0008	90.4653	0.0008	$P2_1/n$ (14)
JB1015A_26_500	463	6	12.4270	0.0008	8.9459	0.0008	8.8321	0.0008	90.4898	0.0008	$P2_1/n$ (14)
JB1015A_27_400	395	6	12.4275	0.0008	8.9428	0.0008	8.8308	0.0008	90.5046	0.0008	$P2_1/n$ (14)
JB1015A_28_300	316	6	12.4278	0.0008	8.9383	0.0008	8.8291	0.0008	90.5169	0.0008	$P2_1/n$ (14)
JB1015A_29_220	253	6	12.4282	0.0008	8.9342	0.0008	8.8277	0.0008	90.5219	0.0008	$P2_1/n$ (14)
JB1015A_30_160	201	6	12.4282	0.0008	8.9307	0.0008	8.8268	0.0008	90.5233	0.0008	$P2_1/n$ (14)
JB1015A_31_25	24	7	12.4292	0.0008	8.9190	0.0008	8.8264	0.0008	90.5160	0.0008	$P2_1/n$ (14)

Table S16 Ca0.5Mg0.5Zr4(PO4)6 unit-cell parameter data

JB1014A APS 17 BM-B: Ca0.5Mg0.5Zr4(PO4)6							
File Name	Temperature (°C)	Temperature ESD (°C)	*a*-Axis Unit-cell Parameter (Å)	*a*-Axis Unit-cell Parameter ESD (Å)	*c*-Axis Unit-cell Parameter (Å)	*c*-Axis Unit-cell Parameter ESD (Å)	Space Group
JB1014A_01_25	25	26	8.7699	0.0004	22.7476	0.0004	?
JB1014A_02_25	23	25	8.7701	0.0004	22.7473	0.0004	?
JB1014A_03_200	303	15	8.7686	0.0003	22.7780	0.0003	?
JB1014A_04_300	386	12	8.7697	0.0003	22.7847	0.0003	?
JB1014A_05_400	449	9	8.7713	0.0002	22.7776	0.0002	R-3 (148)
JB1014A_06_500	514	6	8.7736	0.0001	22.7664	0.0001	R-3

							(148)
JB1014A_07_600	575	5	8.7747	0.0001	22.7644	0.0001	R-3 (148)
JB1014A_08_650	612	5	8.7746	0.0001	22.7694	0.0001	R-3 (148)
JB1014A_09_700	649	5	8.7745	0.0001	22.7750	0.0001	R-3 (148)
JB1014A_10_750	684	5	8.7743	0.0001	22.7803	0.0001	R-3 (148)
JB1014A_11_800	721	5	8.7743	0.0001	22.7859	0.0001	R-3 (148)
JB1014A_12_850	756	5	8.7742	0.0001	22.7916	0.0001	R-3 (148)
JB1014A_13_900	801	5	8.7743	0.0001	22.7985	0.0001	R-3 (148)
JB1014A_14_925	820	5	8.7742	0.0001	22.8021	0.0001	R-3 (148)
JB1014A_15_950	847	4	8.7743	0.0001	22.8067	0.0001	R-3 (148)
JB1014A_16_975	868	5	8.7743	0.0001	22.8108	0.0001	R-3 (148)
JB1014A_17_1000	893	5	8.7743	0.0001	22.8154	0.0001	R-3 (148)
JB1014A_18_1025	918	5	8.7745	0.0001	22.8202	0.0001	R-3 (148)
JB1014A_19_1050	942	4	8.7745	0.0001	22.8253	0.0001	R-3 (148)
JB1014A_20_1000	905	4	8.7742	0.0001	22.8188	0.0001	R-3 (148)
JB1014A_21_950	866	5	8.7741	0.0001	22.8117	0.0001	R-3 (148)
JB1014A_22_900	831	5	8.7741	0.0001	22.8049	0.0001	R-3 (148)
JB1014A_23_850	785	5	8.7740	0.0001	22.7976	0.0001	R-3 (148)
JB1014A_24_800	741	5	8.7741	0.0001	22.7906	0.0001	R-3 (148)
JB1014A_25_750	695	6	8.7742	0.0001	22.7838	0.0001	R-3 (148)
JB1014A_26_700	648	6	8.7743	0.0001	22.7773	0.0001	R-3 (148)
JB1014A_27_650	605	6	8.7745	0.0001	22.7709	0.0001	R-3 (148)
JB1014A_28_600	559	6	8.7746	0.0001	22.7650	0.0001	R-3 (148)
JB1014A_29_500	486	7	8.7729	0.0002	22.7698	0.0002	R-3 (148)
JB1014A_30_400	425	12	8.7707	0.0002	22.7803	0.0002	R-3 (148)
JB1014A_31_300	341	16	8.7695	0.0003	22.7767	0.0003	?
JB1014A_32_200	255	19	8.7685	0.0003	22.7644	0.0003	?
JB1014A_33_25	22	9	8.7701	0.0004	22.7438	0.0004	?
